# Exfoliated 2D Nanosheet‐Based Conjugated Polymer Composites with P‐N Heterojunction Interfaces for Highly Efficient Electrocatalytic Hydrogen Evolution

**DOI:** 10.1002/advs.202407061

**Published:** 2024-07-31

**Authors:** Cheng‐Yu Tsai, Hsu‐Sheng Li, Kumasser Kusse Kuchayita, Hsin‐Chih Huang, Wei‐Nien Su, Chih‐Chia Cheng

**Affiliations:** ^1^ Graduate Institute of Applied Science and Technology National Taiwan University of Science and Technology Taipei 10607 Taiwan; ^2^ Department of Materials Science and Engineering National Formosa University Yunlin 63201 Taiwan; ^3^ Advanced Membrane Materials Research Center National Taiwan University of Science and Technology Taipei 10607 Taiwan

**Keywords:** exfoliated molybdenum disulfide nanosheets, hydrogen evolution reaction, Iodide oxidation reaction, multifunctional composites, non‐noble metal‐based electrochemical catalysts, P‐N heterojunction interfaces

## Abstract

We have achieved a significant breakthrough in the preparation and development of two‐dimensional nanocomposites with P‐N heterojunction interfaces as efficient cathode catalysts for electrochemical hydrogen evolution reaction (HER) and iodide oxidation reaction (IOR). P‐type acid‐doped polyaniline (PANI) and N‐type exfoliated molybdenum disulfide (MoS_2_) nanosheets can form structurally stable composites due to formation of P‐N heterojunction structures at their interfaces. These P‐N heterojunctions facilitate charge transfer from PANI to MoS_2_ structures and thus significantly enhance the catalytic efficiency of MoS_2_ in the HER and IOR. Herein, by combining efficient sodium‐functionalized chitosan‐assisted MoS_2_ exfoliation, electropolymerization of PANI on nickel foam (NF) substrate, and electrochemical activation, controllable and scalable Na‐Chitosan/MoS_2_/PANI/NF electrodes are successfully constructed as non‐noble metal‐based electrochemical catalysts. Compared to a commercial platinum/carbon (Pt/C) catalyst, the Na‐Chitosan/MoS_2_/PANI/NF electrode exhibits significantly lower resistance and overpotential, a similar Tafel slope, and excellent catalytic stability at high current densities, demonstrating excellent catalytic performance in the HER under acidic conditions. More importantly, results obtained from proton exchange membrane fuel cell devices confirm the Na‐Chitosan/MoS_2_/PANI/NF electrode exhibits a low turn‐on voltage, high current density, and stable operation at 2 V. Thus, this system holds potential as a replacement for Pt/C with feasibility for applications in energy‐related fields.

## Graphical abstract

Compared to commercial noble‐metal‐based platinum/carbon electrocatalysts, an emerging organic/inorganic heterojunction material system is presented that demonstrates outstanding enhancements in electrochemical catalytic capability and structural stability due to the formation of P‐N heterojunction interfaces between N‐type exfoliated MoS_2_ nanosheets and P‐type polyaniline. This system exhibits promising performance and stable operation in fuel cell devices, confirming its potential for application in various catalytic and energy fields.

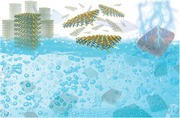

## 1 Introduction

The current global energy crisis and environmental degradation are primarily attributed to the extensive extraction of traditional fossil fuels, highlighting the urgent need to transition to renewable energy sources such as biomass, solar, wind, and hydroelectric power. The cost‐effectiveness of these alternative solutions is increasing, which will be crucial to reducing greenhouse gas emissions and addressing climate change. To meet the urgent demand for sustainable energy storage and conversion systems, several research teams are paying close attention to alternative energy sources such as fuel cells, electrochemical water splitting, and metal‐air batteries, as these technologies offer high energy densities and environmentally friendly characteristics.^[^ ^1^, ^2^, ^3^ ^]^ Hydrogen is considered an economically efficient, clean fuel with tremendous potential because it is abundant, non‐toxic, and only emits water during its reaction and conversion.^[^ ^4^ ^]^ However, current industrial methods for hydrogen production using fossil fuels lead to significant greenhouse gas emissions. The use of renewable energy, such as water electrolysis, will play a crucial role in the transition away from fossil fuel energy sources. Electrochemical water splitting, particularly if facilitated by efficient catalysts, offers a sustainable pathway for hydrogen production. Among the potential catalysts for the production of hydrogen, commercial noble metal platinum‐group catalysts are the most well‐characterized and have excellent catalytic performance, but cost and scarcity issues impede their future development.^[^ ^5^, ^6^ ^]^ Therefore, researchers from various fields are actively seeking potential alternatives to platinum‐based catalysts. However, significant challenges limit the utilization of readily available and stable materials to develop efficient electrocatalysts and thus hinder the achievement of cost‐effectiveness, stability, and purity in industrial‐scale hydrogen production through water electrolysis.^[^ ^7^ ^]^

Transition metal dichalcogenides (TMDs) are a class of 2D layered materials characterized by X─M─X bonds, where M is a transition metal and X = O, S, Se, or Te, primarily held together by van der Waals forces.^[^ ^8^ ^]^ TMDs, especially molybdenum disulfide (MoS_2_), have garnered widespread attention due to their abundance on Earth, excellent structural stability, and electrocatalytic activity, particularly in the hydrogen evolution reaction (HER). Recent studies have highlighted the immense potential of MoS_2_ as an efficient catalyst and electrode material in various energy storage and production applications, including batteries and solar cells.^[^ ^8^ ^]^ The two key factors that influence the catalytic performance of MoS_2_ are charge transfer kinetics and effective active sites. Therefore, approaches such as phase transitions, doping, formation of heterostructures, and preparation of composites with conductive materials have been explored to enhance the catalytic performance of MoS_2_.^[^ ^9^, ^10^ ^]^ For example, Cui and co‐workers developed stable MoS_2_ thin films with vertically aligned layers, which allows the edges to be optimally exposed on the surface.^[^ ^11^ ^]^ Despite their relatively large Tafel slopes, these edge‐terminated MoS_2_ films possess excellent HER exchange current densities. Xie and co‐workers developed defect‐rich MoS_2_ structures by varying the precursor concentration and thiourea content.^[^ ^12^ ^]^ These defect‐rich MoS_2_ nanosheets exhibit excellent HER performance, with an onset potential of 0.12 V and a Tafel slope of 50 mV dec^−1^. Similarly, Sun and co‐workers found that semimetallic vanadium‐doped MoS_2_ nanosheets exhibited enhanced electrical conductivity, and thereby improved catalytic activity for the HER.^[^ ^13^ ^]^ Although these approaches effectively enhance the catalytic performance of MoS_2_, a performance gap still exists compared to platinum‐based catalysts. Therefore, an urgent challenge remains in finding ways to efficiently evolve the catalytic potential of MoS_2_.

P‐N heterojunction catalysts are formed by closely contacting two different types (P‐type and N‐type) of semiconductor materials to create heterojunction interfaces.^[^ ^14^ ^]^ When applied to catalyze the HER, the P‐N heterogeneous junction structure offers several advantages, including facilitating efficient charge separation and migration, reducing recombination losses by charge carriers, and increasing the surface area available for catalytic reactions. Compared to single‐component catalysts, these features collectively enhance the catalytic activity and efficiency of the HER.^[^ ^15^ ^]^ However, the development of P‐N heterojunction catalysts still predominantly relies on noble metal‐based materials, which impose considerable challenges and development limitations in practical applications.^[^ ^16^ ^]^ Inspired by the concept of P‐N heterojunction catalysts, we hypothesized that P‐N heterojunction catalysis could potentially enhance the catalytic properties and structural stability of MoS_2_. Based on our understanding, MoS_2_ with sulfur vacancies is a typical N‐type semiconductor material, meaning its junctions inherently carry positive charge and can conduct or accept charge carriers.^[^ ^17^ ^]^ Polyaniline (PANI) is a fascinating conjugated polymer that can exhibit both N‐type and P‐type behavior, depending on its doping level and specific conditions.^[^ ^18^ ^]^ Generally, when PANI is heavily doped with electron‐rich substances such as halogen elements, it transitions to an N‐type semiconductor. On the contrary, when PANI is doped with electron‐deficient substances such as sulfuric acid or nitric acid, it transitions to a P‐type semiconductor. In the P‐type state, PANI has an excess of positive charge carriers (holes) due to the removal of electrons, which contributes to an increase in conductivity. Thus, we confidently propose that through appropriate exfoliation processes and electropolymerization pathways, the combination of N‐type exfoliated MoS_2_ nanosheets with P‐type acid‐doped PANI may lead to a novel organic‐inorganic P‐N heterojunction catalytic system. We envisioned that this system could not only facilitate charge transfer between PANI and MoS_2_ to enhance the structural stability and overall conductivity of MoS_2_ nanosheets, but also significantly increase the catalytic surface area and HER catalytic performance of MoS_2_. In addition, the development of this strategy may unveil further opportunities for applications in various catalytic and energy‐related fields.

**Scheme 1:**
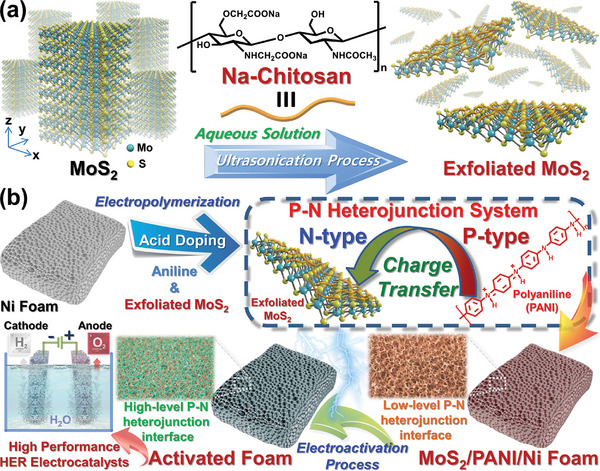
a) Schematic illustration of the process of efficient exfoliation of MoS_2_ crystals assisted by sodium‐functionalized Na‐Chitosan to form water‐dispersible and few‐layered exfoliated MoS_2_ nanosheets. b) Construction of Na‐Chitosan/MoS_2_/PANI/NF electrodes with P‐N heterojunction interfaces and excellent electrochemical HER catalytic capability through the processes of electropolymerization and electrochemical activation. The dashed frame illustrates the formation of stable charge transfer between P‐type PANI and N‐type exfoliated MoS_2_ nanosheets.

Thus, we aimed to develop a highly promising strategy to prepare MoS_2_/PANI composites with a P‐N heterojunction interface and excellent HER catalytic capability by combining exfoliated N‐type MoS_2_ nanosheets with P‐type PANI. Based on the approach of efficient sodium‐functionalized chitosan (Na‐Chitosan) assisted MoS_2_ exfoliation, we could easily fabricate exfoliated MoS_2_ nanosheets with high solid content, long‐term dispersion stability, and controlled layer number and physical properties in water. The freeze‐dried exfoliated MoS_2_ nanosheets can be stably preserved and also easily re‐dispersed in water, indicating that Na‐Chitosan, as a unique dispersing agent, provides an effective and reliable method to construct water‐dispersed and multifunctional 2D nanocomposites. Subsequently, due to the P‐type and N‐type semiconductor properties of PANI and MoS_2_ respectively, the exfoliated MoS_2_ nanosheets were not only successfully incorporated into the PANI matrix through electropolymerization (EP) on a nickel foam (NF) substrate, but could undergo electrochemical activation (EA) leading to stable formation of a P‐N heterojunction interface between PANI and MoS_2_ (**Scheme** **1**). Ultimately, compared to a commercial carbon‐supported platinum catalyst (Pt/C), the resulting MoS_2_/PANI/NF composites exhibit significantly lower resistance and overpotential, a comparable Tafel slope, and excellent long‐term catalytic stability in the electrochemical HER, indicating this non‐noble metal‐based electrochemical catalytic system demonstrates excellent HER catalytic performance. More importantly, analysis of the HER and iodide oxidation reaction (IOR) in proton exchange membrane fuel cell (PEMFC) devices confirmed that the MoS_2_/PANI/NF composites, serving as a cathode electrode catalyst, exhibit a low turn‐on voltage, high current density, and stable power output. Thus, the MoS_2_/PANI/NF system can efficiently produce hydrogen and promote the stable operation of PEMFC devices and also holds the potential to replace Pt/C and be applied in various catalytic and energy fields. As far as we know, this is the first study to propose the use of exfoliated 2D semiconductor nanosheets combined with conjugated polymers to develop efficient electrocatalytic materials with P‐N heterojunction interfaces. Overall, the newly discovered catalytic system described in this work may provide a simple, reliable approach to achieving high‐efficiency alternative energy sources.

## 2 Results and Discussion

We propose an efficient and simple approach to prepare few‐layered MoS_2_ nanosheets at high yield via liquid‐phase exfoliation of pristine MoS_2_ bulk crystals in water using synthetic sodium‐functionalized chitosan biopolymer (Na‐Chitosan), which is achieved by the formation of specific interactions between Na‐Chitosan and the surface of MoS_2_ (Scheme 1a). Although MoS_2_ crystals exhibit low solubility or dispersibility in aqueous solutions,^[^ ^19^ ^]^ the sodium ions, amino, and amide groups on the side chains of Na‐Chitosan facilitate non‐covalent interactions with the surface of MoS_2_, thus leading to the attachment of Na‐Chitosan onto the surface of MoS_2_ and the formation of exfoliated MoS_2_ nanosheets in water. The nanosheets have well‐controlled structural and physical properties, primarily depending on the amount of Na‐Chitosan present in the solution. Na‐Chitosan polymer was synthesized by reacting methyl chloroacetate and commercial chitosan powder in a mixture of aqueous sodium hydroxide and isopropanol at 65 °C for 4 h. Subsequently, distillation, retreatment with sodium hydroxide, dialysis, and freeze‐drying were employed to produce white spongy Na‐Chitosan at a yield of 65% (Figure S1a, Supporting Information). MoS_2_ crystals were mixed with different amounts of Na‐Chitosan in aqueous solution, followed by ultrasonication under ice bath conditions for 30 min. Eventually, water‐dispersible MoS_2_ nanosheets were obtained by centrifugation. Before investigating the water dispersibility and physical properties of MoS_2_ nanosheets produced by liquid‐phase exfoliation assisted by Na‐Chitosan, we first explored the chemical structure of Na‐Chitosan and its self‐assembly behavior in aqueous solution. These explorations aimed to confirm the co‐assembly process of MoS_2_ and Na‐Chitosan in water and the mechanism of formation of few‐layered exfoliated MoS_2_ nanosheets in water, with the objectives of validating the reliability and controllability of this newly developed MoS_2_ exfoliation process and to potentially promote its application across various fields.

We assessed the structure and molecular weight distribution of Na‐Chitosan using Fourier‐transform infrared spectroscopy (FTIR), proton and carbon nuclear magnetic resonance spectroscopy (^1^H and ^13^C NMR), and water‐based gel permeation chromatography (GPC). FTIR spectra (Figure S2, Supporting Information) confirmed the presence of characteristic peaks for symmetric and asymmetric stretching vibrations of the carboxylate ions (─COO^−^) in the structure of Na‐Chitosan (located at 1594 and 1409 cm^−1^, respectively). ^1^H and ^13^C NMR spectra (Figures S3 and S4, Supporting Information) successfully confirmed all characteristic functional groups in the Na‐Chitosan structure. Through integration analysis in Figure S4a (Supporting Information) and calculation of the degree of deacetylation (DD) and degree of substitution (DS) [i.e., the total degree of carboxymethyl substitution on O‐C6 (*f6*), O‐C3 (*f3*), and N‐C2 (*f2*) in Table S1, Supporting Information],^[^ ^20^ ^]^ the DD and DS of Na‐Chitosan were determined to be 97.8% and 1.12, respectively. Furthermore, water‐based GPC (Figure S5, Supporting Information) demonstrated that Na‐Chitosan has a high molecular weight [weight average molecular weight (*M*_w_) = 216900] and a narrow molecular weight distribution [polydispersity index (PDI) = 1.29]. Collectively, these results demonstrate the successful synthesis of Na‐Chitosan polymer with high molecular weight and structural uniformity. The introduction of ionic groups significantly increases the solubility of the polymer structure in both organic solvents and aqueous solutions. Therefore, we preliminarily evaluated the solubility of chitosan and Na‐Chitosan. Chitosan has poor solubility in typical organic solvents and aqueous media. However, Na‐Chitosan obtained by introducing sodium acetate groups into the chitosan side chain not only exhibits high solubility in highly polar organic solvents such as dimethyl sulfoxide and dimethylformamide, but also readily dissolves in water — even at concentrations as high as 50 mg mL^−1^. These results demonstrated the significant impact of the presence of sodium acetate groups on the solubility of chitosan (see Figure S1b, Supporting Information) and further inspired us to explore the self‐assembly behavior of highly water‐soluble Na‐Chitosan in aqueous solution.

First, we used pyrene as a fluorescent probe to determine the critical micelle concentration (CMC) of Na‐Chitosan through photoluminescence (PL) spectroscopy.^[^ ^21^ ^]^ As determined by the inflection point on the curve in Figure S6 (Supporting Information), the CMC value of Na‐Chitosan is 0.0056 mg mL^−1^, clearly demonstrating that the hydrophilic sodium acetate groups introduced into the side chains of chitosan significantly enhance the aggregation or entanglement of the polymer in aqueous solutions.^[^ ^22^ ^]^ In other words, the sodium acetate groups significantly facilitate interactions between polymer chains, thereby resulting in a low CMC value. Next, we further investigated the self‐assembly structure and morphology of Na‐Chitosan in aqueous solution through dynamic light scattering (DLS), zeta potential (ζ), atomic force microscopy (AFM), and scanning electron microscopy (SEM) analyses. As shown in Figure S7a (Supporting Information), at a concentration of 1.0 mg mL^−1^ of Na‐Chitosan (above its CMC), DLS revealed that Na‐Chitosan exhibits a large aggregate structure, with an average hydrodynamic size of 685.1 ± 162.5 nm [ζ = −33.18 ± 1.26 mV]. This indicates that Na‐Chitosan spontaneously forms nanoobjects with a high degree of aggregation in aqueous solution, possibly due to electrostatic interactions between the sodium acetate moieties and the repulsive forces between the main chain and hydrophilic sodium acetate side chains of Na‐Chitosan.^[^ ^23^ ^]^ In validation of these results, AFM and SEM images (Figure S7b–d, Supporting Information) confirmed that Na‐Chitosan forms approximately spherical structures with irregular surfaces and average sizes ranging widely from several hundred nanometers to 1 µm. Collectively, these findings clearly confirm that the presence of sodium acetate moieties within the Na‐Chitosan structure enhances its water‐solubility overall, and also serves as a crucial factor leading to the formation of self‐assembled nanospheres in aqueous environments.

Next, we systematically investigated how the addition of different amounts of Na‐Chitosan affects the formation of exfoliated MoS_2_ nanosheets in water and their related physical properties. First, to confirm the optimal preparation ratio of Na‐Chitosan/MoS_2_ dispersion solution, we added 5 mg of MoS_2_ crystals into Na‐Chitosan aqueous solutions ranging from 0.1 to 10 mg mL^−1^, followed by ultrasonication and centrifugation. The resulting Na‐Chitosan/MoS_2_ dispersion solutions were analyzed to determine the MoS_2_ solid content and surface charge. As shown in Figure S8 (Supporting Information), the content of exfoliated MoS_2_ nanosheets in the aqueous solution increased with the concentration of Na‐Chitosan added. The highest MoS_2_ content of 1.85 mg mL^−1^ was achieved at a Na‐Chitosan concentration of 5 mg mL^−1^. However, as the Na‐Chitosan concentration was further increased to 10 mg mL^−1^, the MoS_2_ content of the solution significantly decreased to 0.46 mg mL^−1^. Thus, an excess of Na‐Chitosan (exceeding 5 mg mL^−1^) may lead to the formation of aggregates in the solution, and thus significantly decrease the exfoliation efficiency of MoS_2_. A similar trend was also observed in the analysis of surface charge (Figure S9, Supporting Information); the negative ζ values of the Na‐Chitosan/MoS_2_ dispersion gradually decreased as the Na‐Chitosan concentration increased, suggesting that increasing the Na‐Chitosan concentration gradually decreased the stability of the exfoliated MoS_2_ nanosheets in aqueous solution.^[^ ^24^ ^]^ Based on the content and ζ values of exfoliated MoS_2_ in an aqueous solution (Figures S8 and S9, Supporting Information) and to achieve the optimal compromise between yield and structural stability, for subsequent investigations, we prepared exfoliated MoS_2_ dispersions using 1 mg mL^−1^ aqueous Na‐Chitosan and MoS_2_ crystals at 1, 3, and 5 mg mL^−1^, respectively; the resulting nanocomposites will henceforth be referred to as 1/1, 1/3, and 1/5 Na‐Chitosan/MoS_2_, respectively.

**Figure 1:**
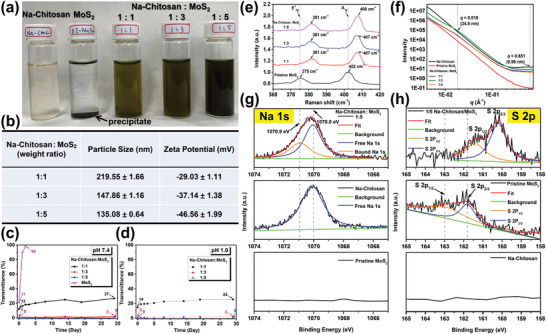
a) Photograph of aqueous solutions of original Na‐Chitosan, pristine MoS_2_, and Na‐Chitosan/MoS_2_ at different ratios (1/1, 1/3, and 1/5). b) Compilation of particle size and zeta potential values for 1/1, 1/3, and 1/5 Na‐Chitosan/MoS_2_ aqueous solutions. Changes in optical transmittance (at 500 nm) of 1/1, 1/3, and 1/5 Na‐Chitosan/MoS_2_ in water at c) pH 7.4 or d) pH 1.0 at 25 °C over 29 d. e) Raman spectra of pristine MoS_2_, 1/1, 1/3, and 1/5 Na‐Chitosan/MoS_2_ composites recorded at 25 °C. f) SAXS patterns of pristine MoS_2_, Na‐Chitosan, and Na‐Chitosan/MoS_2_ composites at different ratios (1/1, 1/3, and 1/5) were obtained at 25 °C. XPS spectra of g) Na 1s and h) S 2p of pristine MoS_2_, Na‐Chitosan, and 1/5 Na‐Chitosan/MoS_2_ composites.

After ultrasonication and centrifugation, the macroscopic darkness of the resulting Na‐Chitosan/MoS_2_ solution gradually increased with the content of exfoliated MoS_2_ (**Figure** **1**a; Figure S8, Supporting Information). Specifically, the 1/5 Na‐Chitosan/MoS_2_ composition resulted in a completely opaque black solution, whereas the 1/1 Na‐Chitosan/MoS_2_ composition appeared relatively transparent with a brownish color, suggesting that the 1/5 Na‐Chitosan/MoS_2_ composition contains the optimal amount of Na‐Chitosan to assist the formation of and stabilize exfoliated MoS_2_ nanosheets in water. In contrast, excess Na‐Chitosan (as in the 1/1 Na‐Chitosan/MoS_2_ composition) led to the formation of self‐aggregates of Na‐Chitosan, which reduced the efficiency and yield of MoS_2_ exfoliation. Nevertheless, even after ultrasonication, the pristine MoS_2_ crystals still completely precipitated in water (Figure 1a), clearly indicating the indispensable role of Na‐Chitosan in the exfoliation process of MoS_2_ crystals and formation of stably dispersed nanosheets in water. Subsequently, to further confirm the influence of the content of Na‐Chitosan, DLS, and surface charge measurements were performed to assess the size distribution and dispersion stability. As depicted in Figure 1b, the 1/5 Na‐Chitosan/MoS_2_ solution exhibited the lowest average hydrodynamic size (135.08 ± 0.64 nm) and the highest negative ζ value (−46.56 ± 1.99 mV), whereas the 1/1 Na‐Chitosan/MoS_2_ solution had the largest average hydrodynamic size (219.55 ± 1.66 nm) and smallest negative ζ value (−29.03 ± 1.11 mV). This confirmed that the 1/5 Na‐Chitosan/MoS_2_ composite contains the optimal amount of Na‐Chitosan, leading to the formation of minimally sized MoS_2_ nanosheets with high stability, while effectively avoiding the adverse effects of excess Na‐Chitosan. Notably, compared to pristine Na‐Chitosan in water, no significant large‐scale aggregation was observed for any of the three composites in water, indicating a strong affinity between Na‐Chitosan and the surface of MoS_2_ effectively inhibits the formation of self‐aggregates of Na‐Chitosan in solution (Figure S7, Supporting Information). In other words, the polymer chains of Na‐Chitosan tightly adhere to the surface of the exfoliated MoS_2_ nanosheets, thereby stabilizing their dispersion in water.^[^ ^25^ ^]^ These unique findings sparked our curiosity to investigate the long‐term stability of Na‐Chitosan/MoS_2_ nanosheets under different aqueous conditions.

Thus, we further evaluated the long‐term dispersion stability of pristine MoS_2_ and Na‐Chitosan/MoS_2_ solutions in normal and acidic aqueous environments at 25 °C by monitoring the percentage transmittance at 500 nm over time using UV–vis spectroscopy. The transmittance of the pristine MoS_2_ aqueous solution rapidly increased from 22% to 99% after 2 d of monitoring (Figure 1c; Figure S10a, Supporting Information), indicating extremely low compatibility between pristine MoS_2_ and the aqueous environment led to complete precipitation within 2 d. In contrast to pristine MoS_2_, the three solutions with different ratios of Na‐Chitosan/MoS_2_ exhibited significantly superior long‐term MoS_2_ dispersion stability (Figure 1c), underscoring once again the substantial contribution of Na‐Chitosan to the exfoliation process and structural stability of MoS_2_. Surprisingly, the transmittance values of the 1/3 and 1/5 Na‐Chitosan/MoS_2_ solutions remained almost unchanged over 29 d, at 0% and 2% respectively (Figure 1c; Figure S10c,d, Supporting Information). This indicates the presence of optimal contents of Na‐Chitosan in these two solutions, thereby achieving both stable suspension of exfoliated MoS_2_ nanosheets in water and significantly enhanced long‐term stability. In contrast, the transmittance values of the 1/1 Na‐Chitosan/MoS_2_ solution increased from 12% to 27% after 29 d (Figure 1c; Figure S10b, Supporting Information), suggesting that excess of Na‐Chitosan on the surface of MoS_2_ nanosheets may accelerate aggregation and thereby reduce the dispersion stability of the nanosheets in water. The same trends were also observed under acidic conditions in pH 1.0 hydrogen chloride (Figure 1d; Figure S11, Supporting Information). Overall, these results further confirm the excellent long‐term dispersion stability of Na‐Chitosan/MoS_2_ solutions and also demonstrate their high resistance to highly acidic environments, indicting potential for applications in various fields.^[^ ^26^, ^27^ ^]^

The results above confirmed that the affinity between Na‐Chitosan and the surface of MoS_2_ plays a crucial role in facilitating the exfoliation of MoS_2_ crystals. Therefore, to verify the impact of the affinity between the two materials, Raman spectroscopy and small‐ and wide‐angle X‐ray scattering (SAXS and WAXS) were conducted at 25 °C to directly observe the transformation of the microstructure of Na‐Chitosan/MoS_2_ composites after freeze‐drying. As shown in Figure 1e, the Raman spectrum of the original MoS_2_ crystals exhibited characteristic peaks at 375 and 402 cm^−1^, corresponding to in‐plane vibration ($E_{2g}^{1}$) of sulfur/molybdenum atoms and out‐of‐plane vibration ($A_{g}^{1}$) of sulfur atoms, respectively.^[^ ^28^ ^]^ Interestingly, in the presence of Na‐Chitosan, a significant blue shift was observed in both the $E_{2g}^{1}$ and $A_{g}^{1}$ peaks of all Na‐Chitosan/MoS_2_ composites, shifting to ≈381 and 408 cm^−1^, respectively, suggesting transformation of the original MoS_2_ crystal structure into highly disordered nanostructures.^[^ ^25^, ^29^ ^]^ Furthermore, the spectra of all Na‐Chitosan/MoS_2_ composites exhibited similar results, indicating that regardless of the amount of Na‐Chitosan added, the exfoliated MoS_2_ nanosheets possessed similar structural characteristics after centrifugation. This result also reaffirms that the 1/5 Na‐Chitosan/MoS_2_ composites (prepared with the optimized Na‐Chitosan content) not only achieve the highest yield of exfoliated MoS_2_ nanosheets, but also exhibit superior long‐term dispersion stability. The SAXS patterns in Figure 1f also revealed structural changes in Na‐Chitosan/MoS_2_. Na‐Chitosan exhibited a characteristic peak at a high *q* region with a *d*‐spacing of 0.96 nm (*q* = 0.651 Å^−1^), which can be attributed to the intermolecular distance between the sodium acetate side groups, which form a bilayer structure; namely, the distance between polymer chains.^[^ ^30^ ^]^ Unexpectedly, compared to the original Na‐Chitosan, all Na‐Chitosan/MoS_2_ composites exhibited a broad peak in the low *q* region with a size of ≈36.8 nm (*q* = 0.018 Å^−1^). Meanwhile, the characteristic peak of Na‐Chitosan at 0.96 nm completely disappeared. This reveals the presence of specific forces (or affinity) between Na‐Chitosan and the surface of MoS_2_ effectively disrupts the structure of the Na‐Chitosan aggregates and instead promotes the formation of specific self‐assembled structures of Na‐Chitosan on the surface of MoS_2_. The formation of this structure can be inferred to correlate with the presence of semiconductor 2H and metallic 1T phases on the exfoliated MoS_2_ nanosheets.^[^ ^31^ ^]^ In other words, the formation of the broad peak at *q* = 0.018 Å^−1^ may be attributed to varying degrees of non‐covalent interactions between the polymer chains of Na‐Chitosan and the non‐active semiconducting 2H phase and highly active metallic 1T phase on the MoS_2_ nanosheets.^[^ ^25^, ^32^ ^]^ Subsequently, Na‐Chitosan forms large‐scale self‐assembled structures at this interface. In addition, the WAXS pattern in the high *q* region (*q* = 0.5‐5.0 Å^−1^) also further demonstrates that the Na‐Chitosan content of the Na‐Chitosan/MoS_2_ composites can be modulated to achieve the desired yield of exfoliated MoS_2_ nanosheets. As presented in Figure S12 (Supporting Information), in the spectra of the original MoS_2_ and Na‐Chitosan/MoS_2_ composites with different compositions, the intensity of the (002) signal peak at 1.025 Å^−1^ decreased gradually as the Na‐Chitosan content was reduced. This peak nearly disappeared and no other crystalline signal peaks at higher *q* values were observed in the pattern for 1/5 Na‐Chitosan/MoS_2_, indicating that the structure had transitioned from the originally highly stacked MoS_2_ crystals to a completely disordered and few‐layered amorphous‐like structure.^[^ ^33^ ^]^ These results further confirm that strong non‐covalent interactions between the polymer chains of Na‐Chitosan and the surface of MoS_2_ promote the adsorption of the polymer chains onto the surface of the nanosheets and the formation of specific aggregate structures, and also effectively confer stable dispersion of the exfoliated nanosheets in water.

**Figure 2:**
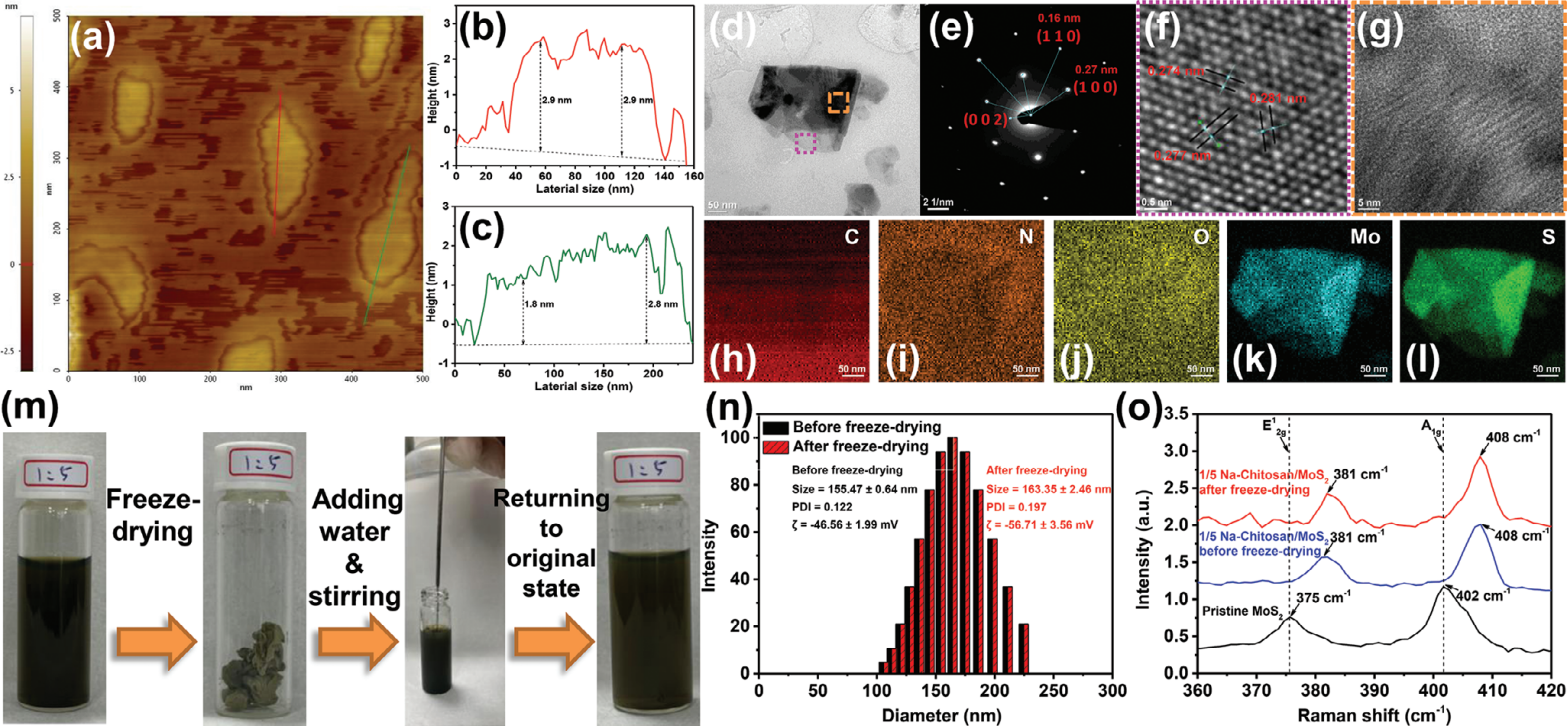
a) AFM images and b,c) topographical profiles of 1/5 Na‐Chitosan/MoS_2_ composites recorded at 25 °C. Line contours in (b) and (c), respectively, correspond to the red and green lines drawn within the AFM image in (a), representing the lateral and height distributions of 1/5 Na‐Chitosan/MoS_2_ nanosheets. d) HRTEM image and e) corresponding SAED pattern of the 1/5 Na‐Chitosan/MoS_2_ composites. f,g) HRTEM images of the region enclosed by the pink and orange dashed frames in (d). The black lines plotted in (f) were used to calculate the spacing between the (100) crystal planes. For the HRTEM image in (d), corresponding elemental mapping images for h) C, i) N, j) O, k) Mo, and l) S elements of 1/5 Na‐Chitosan/MoS_2_ composites. m) Process of re‐dispersion of freeze‐dried 1/5 Na‐Chitosan/MoS_2_ solid in water. n) Particle sizes, zeta potential values, and o) Raman spectra of 1/5 Na‐Chitosan/MoS_2_ aqueous solution before and after freeze‐drying.

X‐ray photoelectron spectroscopy (XPS) was employed to provide additional evidence and confirm the specific interaction between Na‐Chitosan and MoS_2_ in the solid state (Figure S13, Supporting Information). In the original Na‐Chitosan spectrum in Figure 1g, a single distinct peak for Na 1s is observed at 1070.0 eV. In comparison, a newly generated peak belonging to the Na‐bound state (located at 1070.9 eV) was observed for the 1/5 Na‐Chitosan/MoS_2_ composites, suggesting a specific interaction occurs between the sodium acetate side groups of Na‐Chitosan and the active atomic sites on the surface of MoS_2_.^[^ ^34^ ^]^ This further confirms that the self‐assembled nanostructures that form on the surface of the exfoliated MoS_2_ nanosheets are due to non‐covalent binding with Na‐Chitosan.^[^ ^35^, ^36^ ^]^ In addition, compared to MoS_2_, the characteristic S 2p, S 2s, and Mo 3d peaks in the Na‐Chitosan/MoS_2_ composites shifted toward significantly lower binding energies due to the formation of low‐layer structures (Figure 1h; Figure S14, Supporting Information). Moreover, the characteristic peaks of the oxidized state Mo^6+^ also exhibited significantly higher intensities (Figure S14, Supporting Information), clearly indicating that the exfoliated MoS_2_ nanosheets have more active sites than the original MoS_2_ crystal,^[^ ^36^, ^37^ ^]^ thereby promoting the interaction with Na‐Chitosan and ultimately leading to the formation of exfoliated MoS_2_ nanosheets with structural stability and uniform characteristics. These data not only confirm the spectral characteristics of Na‐Chitosan/MoS_2_ composites, but also further verify the morphology and surface microstructure of the delaminated nanosheets observed by SEM and AFM. The AFM and SEM images in **Figure** **2**a, Figures S15 and S16 (Supporting Information) show that, unlike the original MoS_2_ crystal with micrometer‐sized bulk structures, the 1/5 Na‐Chitosan/MoS_2_ composites exhibit a morphology consisting of uniformly dispersed nanosheets with lateral dimensions ranging from 200 to 300 nm, strongly demonstrating that incorporating Na‐Chitosan into the MoS_2_ solution promotes exfoliation of MoS_2_, thereby obtaining thin‐layered 2D nanosheet‐like structures. Furthermore, the topographic height profiles from AFM and enlarged SEM images (Figure 2b,c; Figure S16b, Supporting Information) also revealed the rough surface features of these exfoliated nanosheets, clearly indicating the presence of self‐assembled aggregates of Na‐chitosan polymer chains adsorbed on the MoS_2_ nanosheets. More importantly, compared to the theoretical thickness of single‐layer MoS_2_ nanosheets (0.615 nm),^[^ ^38^ ^]^ height profile analysis of the AFM images indicates that the thickness of 1/5 Na‐Chitosan/MoS_2_ nanosheets was ≈2.9 nm, suggesting a layer number of less than five (Figure 2b,c) and also certainly confirming the formation of multilayer exfoliated nanosheets.

Due to its thin‐layered nanosheet structure, we further verified the surface microstructure, crystalline characteristics, and compositional distribution of the exfoliated MoS_2_ nanosheets using high‐resolution transmission electron microscopy (HRTEM) and selected‐area electron diffraction (SAED) and elemental mapping measurements. After sample preparation using a cryo‐ultramicrotome, the HRTEM image in Figure 2d clearly shows that the 1/5 Na‐Chitosan/MoS_2_ composites form thin‐layered nanosheet structures with a surface exhibiting a disordered light and dark distribution, suggesting that the surface of the exfoliated MoS_2_ nanosheets is covered (or adsorbed) with some degree of self‐assembled aggregates of Na‐Chitosan. The SAED pattern in Figure 2e further demonstrates that the 1/5 Na‐Chitosan/MoS_2_ composites exhibit a clear hexagonally ordered microstructure with two highly symmetric (100) and (110) spots, while almost no (002) spots are observed. This result aligns with previous SAED patterns of monolayer MoS_2_ nanosheets,^[^ ^39^ ^]^ and also confirms the transformation of the original MoS_2_ crystal into few‐layered nanosheets. In addition, high‐magnification TEM images (Figure 2d) clearly depicted the microstructure of the bright‐dark regions on the surface of 1/5 Na‐Chitosan/MoS_2_ nanosheets. As shown in Figure 2f (corresponding to the pink dashed frame in Figure 2d), the bright region exhibits a regular (100) crystal plane structure, with an interplanar spacing of ≈0.27–0.28 nm. In contrast, as shown in Figure 2g (corresponding to the orange dashed frame in Figure 2d), the dark region completely lacks the (100) crystal plane structure observed in the bright region. Instead, a layered‐like structure is observed, with interlayer distances ranging from ≈0.5 to 2.0 nm, which may be attributed to the attachment of self‐assembled Na‐Chitosan onto the 1T or 2H phases on the surface of MoS_2_.^[^ ^25^, ^32^ ^]^ This finding further confirms the specific, high‐affinity interactions between the surface of the exfoliated MoS_2_ and Na‐Chitosan. Similarly, elemental mapping analysis (Figure 2h–l) clearly detected the presence of all characteristic elements (C, N, O, Mo, and S) on the exfoliated nanosheets, demonstrating uniform attachment of Na‐Chitosan onto the MoS_2_ nanosheets. Overall, these findings further confirmed that high‐quality and size‐uniform exfoliated MoS_2_ nanosheets can be obtained through this newly developed approach, and also that the yield of exfoliated MoS_2_ nanosheets can be controlled by adjusting the content of Na‐Chitosan.

Compared to the previous literature on water‐dispersible MoS_2_ nanosheets developed so far (Table S2, Supporting Information),^[^ ^40^, ^41^, ^42^, ^43^, ^44^ ^]^ our MoS_2_ exfoliation process assisted by Na‐Chitosan offers a shorter exfoliation processing time (0.5 h) and also achieves the highest exfoliation yield (1.85 mg mL^−1^), lowest size distribution (135 nm), and excellent long‐term dispersion stability. In addition, like Na‐Chitosan, the structurally similar commercial sodium carboxymethylcellulose (Na‐CMC) can produce water‐dispersible MoS_2_ nanosheets under the same processing conditions. However, after 50 d, significant precipitation and a substantial decrease in the ζ value were observed, whereas 1/5 Na‐Chitosan/MoS_2_ maintained the same physical properties (Figure S17, Supporting Information), revealing that the amino and amide groups in the structure of Na‐Chitosan may enhance its affinity and interaction with water molecules, and thereby promote the long‐term stability and dispersion of exfoliated MoS_2_ nanosheets in water. More importantly, the solid‐state 1/5 Na‐Chitosan/MoS_2_ composites could be stably preserved by freeze‐drying and also easily re‐dispersed by the simple addition of water and stirring treatment (Figure 2m; Video S1, Supporting Information). The resulting solutions exhibited very similar hydrodynamic sizes, ζ values, and structural characteristics as the original solution (Figure 2n,o), confirming that Na‐Chitosan was firmly attached to the surface of the exfoliated MoS_2_ nanosheets and imparted the property of stable re‐dispersion in water. These unique and rare sample preservation and re‐preparation characteristics further indicate the wide potential for future development of this newly developed process, and highlight the importance and indispensability of Na‐Chitosan in this process. To confirm the universality of this exfoliation process across different 2D materials, we employed the same processes and evaluation methods to explore the effects of Na‐Chitosan on the exfoliation efficiency and re‐preparation characteristics of a MoS_2_ derivative, molybdenum diselenide (MoSe_2_), and tungsten disulfide (WS_2_) crystals. As shown in Figures S18 and S19 (Supporting Information), these compounds exhibited the same trends as observed for MoS_2_. The addition of Na‐Chitosan enabled the effective dispersal of MoSe_2_ and WS_2_ in water and also allowed for re‐dispersion of the freeze‐dried samples in water, which exhibited similar characteristics to the original solution. This evaluation successfully confirmed the versatility and reliability of Na‐Chitosan to construct water‐dispersible 2D nanomaterials and strongly indicates the potential of Na‐Chitosan to contribute to future developments and applications in environmentally friendly water‐based processes.^[^ ^45^ ^]^

**Figure 3:**
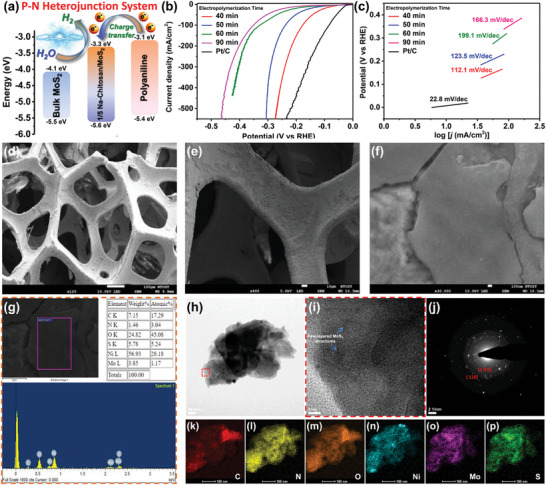
a) *E*_g_ diagrams of bulk MoS_2_, pristine PANI, and 1/5 Na‐Chitosan/MoS_2_ composites, revealing that the 1/5 Na‐Chitosan/MoS_2_ composites can form a P‐N heterojunction system, which probably facilitates charge transfer and catalyzes the electrolysis of water for hydrogen production. HER performance of Na‐Chitosan/MoS_2_/PANI/NF electrodes with different EP times in a 0.5 m H_2_SO_4_ solution: b) LSV curves and c) Tafel plots. SEM images of 1:1 Na‐Chitosan/MoS_2_/PANI/NF electrodes at d) 120x, e) 400x, and f) 30,000x magnification. g) SEM image and corresponding EDX analysis of the Na‐Chitosan/MoS_2_/PANI/NF electrodes. h) HRTEM image of the Na‐Chitosan/MoS_2_/PANI/NF electrodes after the EP process. i) HRTEM images of the region enclosed by the red dashed frame in (h). The blue arrows in (i) indicate the observed few‐layered MoS_2_ structures. For the HRTEM image in (h), corresponding j) SAED pattern and HRTEM/elemental mapping images for k) C, l) N, m) O, n) Ni, and o) Mo and p) S elements of the Na‐Chitosan/MoS_2_/PANI/NF electrodes after the EP process.

Owing to its unique atomic structure, abundance, and low cost, MoS_2_ is a promising negative electrocatalyst for high‐performance hydrogen evolution reaction (HER).^[^ ^36^, ^46^ ^]^ However, compared to traditional noble metal‐based platinum catalysts, there is still significant room for improvement in the HER performance of MoS_2_.^[^ ^47^ ^]^ When produced using our Na‐Chitosan/MoS_2_ system, exfoliated MoS_2_ nanosheets exhibit uniform structural characteristics, high surface area, and dimensional stability. We anticipated that these properties could enhance the catalytic performance of the HER. Although Na‐Chitosan/MoS_2_ nanosheets exhibit high water dispersibility, they may not stably adhere to the electrode surface in the aqueous electrolyte environment required for the HER. Thus, it is necessary to explore other collaborative approaches to enhance their attachment stability on the electrode. Therefore, we propose a potential approach involving the EP of anilinium chloride monomer on a highly conductive nickel foam (NF) substrate. By incorporating 1/5 Na‐Chitosan/MoS_2_ solution into the polymerization reaction, the resulting acid‐doped polyaniline (PANI) thin film can serve as a binder to encapsulate the exfoliated MoS_2_ nanosheets in the matrix. In addition, due to the N‐type and P‐type semiconductor characteristics of MoS_2_ nanosheets and PANI, respectively,^[^ ^17^, ^18^ ^]^ P‐N heterojunction interfaces may form within the PANI matrix, which could significantly improve the attachment stability of MoS_2_ nanosheets and the electrochemical catalytic performance (Scheme 1b). To confirm that the acid‐doped PANI and exfoliated MoS_2_ nanosheets have matching band structures to form a heterojunction interface, we first established their energy band gap (*E*_g_) distribution for the highest occupied molecular orbitals (HOMO) and lowest unoccupied molecular orbitals (LUMO) through cyclic voltammetry and UV‐Vis near‐infrared spectroscopy.^[^ ^48^, ^49^ ^]^ As shown in **Figures** **3**a and S20 (Supporting Information), compared to bulk MoS_2_ with an *E*_g_ of 1.4 eV (with HOMO and LUMO at −5.5 and −4.1 eV, respectively),^[^ ^50^ ^]^ the *E*_g_ of 1/5 Na‐Chitosan/MoS_2_ nanosheets increases to 2.3 eV, demonstrating the lower‐layered structure leads to significant quantum confinement effects, subsequently resulting in a substantial increase in *E*_g_.^[^ ^51^ ^]^ In addition, the 1/5 Na‐Chitosan/MoS_2_ nanosheets exhibited a LUMO value of −3.3 eV, which is close to the LUMO value of PANI (−3.1 eV). This implies that charge transfer can occur rapidly and efficiently within the PANI structure to the Na‐Chitosan/MoS_2_ nanosheets. Consequently, this not only effectively mitigates the detrimental effects of a large energy level mismatch in LUMO, but may also facilitate the formation of stable P‐N heterojunction interfaces between the two materials.

Next, we prepared 1/5 Na‐Chitosan/MoS_2_/PANI composite films on NF substrates through a three‐electrode EP method (Na‐Chitosan/MoS_2_/PANI/NF). The sizes of the resulting samples ranged from 1 cm × 1 cm to 5 cm × 5 cm (Figure S21, Supporting Information), demonstrating that the catalytic materials built through this approach exhibit wide‐range size tunability. Subsequent discussions will focus on the 2 cm × 2 cm Na‐Chitosan/MoS_2_/PANI/NF electrodes. To assess the impact of EP time on the electrochemical HER performance, samples treated for different EP times were directly subjected to linear sweep voltammetry (LSV) in 0.5 m sulfuric acid (H_2_SO_4_) electrolyte solution to establish their corresponding Tafel plots. Since the current stabilizes after 40 min of EP, we compared the results from 40 to 90 min. As shown in Figure 3b,c and Table S3 (Supporting Information), the LSV curves indicate that the overpotential and overall Tafel slope of Na‐Chitosan/MoS_2_/PANI/NF at −10 mA cm^−2^ increase with the EP time, which may be attributed to the undesired effect of a time‐dependent increase in the thickness of PANI. Therefore, the optimal results were achieved at an EP time of 40 min; the Tafel slope and overpotential were 112.1 mV dec^−1^ and 37.8 mV, respectively, which still represent considerable improvements compared to a state‐of‐the‐art commercial Pt/C catalyst (Tafel slope and overpotential of 22.8 mV dec^−1^ and 2.4 mV, respectively). From here onward, samples were prepared under the conditions of EP for 40 min and their physical properties and changes in the NF surface after EP were examined. In comparison to the SEM image of the original NF (Figure S22, Supporting Information), which exhibits a surface with distinct grain boundaries, the surface of Na‐Chitosan/MoS_2_/PANI/NF shows a discontinuous and cracked appearance (Figure 3d–f), implying that the environment during EP may affect the affinity between PANI and the exfoliated MoS_2_ nanosheets. This limitation in forming P‐N heterojunction interfaces may lead to the NF surface displaying a phase‐separated appearance. Analysis of the SEM image in Figure 3g by energy dispersive X‐ray spectroscopy (EDX) revealed that the surface of Na‐Chitosan/MoS_2_/PANI/NF contains all of the expected elemental components, with the MoS_2_ component accounting for ˂10%, reflecting the distribution of MoS_2_ nanosheets within the substrate. Furthermore, HRTEM images and SAED patterns (Figure 3h–j) revealed that the cryo‐ultramicrotome‐treated Na‐Chitosan/MoS_2_/PANI/NF sample exhibited the layer‐like characteristic structures of exfoliated MoS_2_ nanosheets, along with the presence of (100) and (110) spots. Elemental mapping images (Figure 3k–p) also confirmed the presence of all characteristic elements, indicating that—in the composite with NF as the substrate—Na‐Chitosan/MoS_2_ nanosheets were uniformly distributed within the PANI matrix. These results further confirmed that, through the combined pathway of EP and the potential formation of P‐N heterojunction interfaces between MoS_2_ and PANI, exfoliated MoS_2_ nanosheets can be effectively immobilized within PANI, which in turn improves catalytic performance in the electrochemical HER.

**Figure 4:**
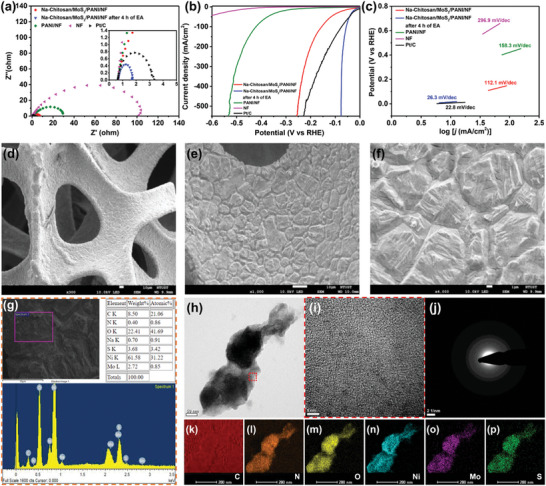
a) EIS Nyquist plots, b) LSV curves, and c) Tafel plots for pristine NF, PANI/NF, Pt/C, and Na‐Chitosan/MoS_2_/PANI/NF electrodes before and after EA treatment in 0.5 m H_2_SO_4_ solution. SEM images at d) 100x, e) 1,000x, and f) 4,000x magnification of the Na‐Chitosan/MoS_2_/PANI/NF electrodes after EA treatment. g) SEM image and corresponding EDX analysis of the Na‐Chitosan/MoS_2_/PANI/NF electrodes after EA treatment. h) HRTEM image of the Na‐Chitosan/MoS_2_/PANI/NF electrodes after EA treatment. i) HRTEM images of the region enclosed by the red dashed frame in (h). For the HRTEM image in (h), corresponding j) SAED pattern and HRTEM/elemental mapping images for k) C, l) N, m) O, n) Ni, and o) Mo and p) S elements in Na‐Chitosan/MoS_2_/PANI/NF electrodes after EA treatment.

Although the EP pathway facilitates the successful incorporation of exfoliated MoS_2_ nanosheets into the PANI matrix, the limited compatibility between their structures results in very restricted formation of P‐N heterojunction interfaces. To overcome this issue, we aimed to promote this interaction and enhance their compatibility by operating in a high‐energy environment, thereby facilitating the formation of highly pronounced P‐N heterojunction interfaces in the matrix and ultimately enhancing the overall electrochemical catalytic efficiency. Therefore, we monitored the process in 0.5 m H_2_SO_4_ solution under a constant current of 500 mA, observing changes in the potential to assess alterations in the catalytic ability of Na‐Chitosan/MoS_2_/PANI/NF. As shown in Figure S23 (Supporting Information), surprisingly, during the 8‐h monitoring period, the potential decreased over time and gradually leveled off after 3 h. This indirectly suggests an improvement in the catalytic efficiency of Na‐Chitosan/MoS_2_/PANI/NF, possibly due to the effective driving of motion between MoS_2_ and PANI at high currents, subsequently promoting the formation of stable P‐N heterojunction interfaces and thus potentially significantly enhancing the efficiency of the HER. This process can be termed an EA procedure. To ensure that the EA treatment reached equilibrium potential, the EA time was set at 4 h, followed by further HER evaluation of the resulting samples. Electrochemical impedance spectroscopy (EIS), LSV, and Tafel plots (**Figure** **4**a–c; Table S3, Supporting Information) revealed that, compared to the results before EA treatment, the Na‐Chitosan/MoS_2_/PANI/NF after EA exhibited a significant decrease in resistance (1.7 ohms), overpotential (14.8 mV at −10 mA cm^−2^), and Tafel slope (26.3 mV dec^−1^), confirming the EA process enhanced the electrochemical catalytic properties of Na‐Chitosan/MoS_2_/PANI/NF. Notably, the overpotential of the EA‐treated Na‐Chitosan/MoS_2_/PANI/NF electrode at a high current density of −500 mA cm^−2^ was only 76.1 mV (Figure 4b), which not only verifies the EIS (Figure 4a), but also indicates that Na‐Chitosan/MoS_2_/PANI/NF can maintain low overpotential characteristics at high current densities due to its excellent conductivity. More importantly, the HER catalytic performance of this newly developed system is comparable to that of Pt/C, indicating the potential of Na‐Chitosan/MoS_2_/PANI/NF to serve as a non‐noble metal electrochemical catalytic system that could possibly replace commercial Pt/C catalysts.

These findings above prompted us to explore the surface morphology and microstructure changes after EA. SEM images (Figure 4d–f) clearly showed that, unlike pre‐EA treatment (Figure 3d–f), the surface of Na‐Chitosan/MoS_2_/PANI/NF exhibited a uniform and solid texture after EA treatment. This indicates that EA treatment promotes the interaction between MoS_2_ and PANI, thereby enhancing their compatibility and the formation of stable P‐N heterojunction interfaces, which ultimately firmly cover the surface of NF. EDX (Figure 4g) detected all expected elemental components; however, compared to pre‐EA EDX (Figure 3g), the MoS_2_ component slightly decreased to 6.4% after EA treatment. This may be due to the EA treatment allowing for more uniform distribution of MoS_2_ nanosheets within the PANI substrate, resulting in slight differences between pre‐ and post‐EA treatment. Similar results were also observed in the HRTEM images and SAED patterns (Figure 4h–j), which confirmed that — following sample preparation with a cryo‐ultramicrotome — Na‐Chitosan/MoS_2_/PANI/NF exhibits a more homogeneous microstructural morphology after EA treatment (Figure 4i) and the characteristic crystal face spots of MoS_2_ nanosheets and NF substrate were not observed (Figure 4j). This indicates the MoS_2_ nanosheets are more uniformly distributed within PANI after EA treatment, and subsequently, the composite adheres more tightly to the surface of NF, resulting in a homogeneous and amorphous‐like microstructure. Moreover, elemental mapping (Figure 4k–p) further confirmed that all characteristic elements were uniformly distributed within the structure, once again verifying that EA treatment promotes the formation of P‐N heterojunction interfaces between MoS_2_ and PANI. Subsequently, these interfaces ultimately facilitate stable charge transfer from PANI to MoS_2_ during HER catalysis, thereby significantly improving the overall HER catalytic efficiency.

**Figure 5:**
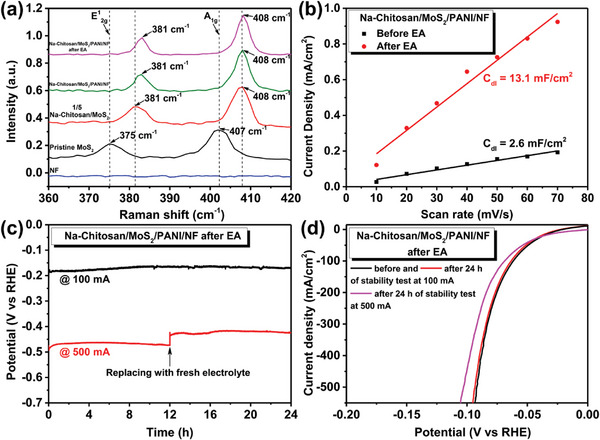
a) Raman spectra of NF, pristine MoS_2_, and Na‐Chitosan/MoS_2_/PANI/NF electrodes after EA treatment. b) *C*_dl_ results for the Na‐Chitosan/MoS_2_/PANI/NF electrodes before and after EA treatment obtained by cyclic voltammetry (CV) over the scanning range of 0.01–0.06 V at various scan rates in 0.5 m H_2_SO_4_ solution. c) Chronopotentiometric stability of the Na‐Chitosan/MoS_2_/PANI/NF electrodes after EA treatment in 0.5 m H_2_SO_4_ solution for 24 h at a fixed current of 100 mA or 500 mA. The potential difference before and after changing the electrolyte is likely due to changes in electrolyte concentration under high current conditions. d) LSV curves for electrochemically activated Na‐Chitosan/MoS_2_/PANI/NF electrodes before and after 24 h of stability testing in 0.5 m H_2_SO_4_ solution at 100 mA or 500 mA.

Raman spectroscopy and XPS were employed to further explore the structural information of Na‐Chitosan/MoS_2_/PANI/NF electrodes after EA treatment. Raman spectra (**Figure** **5**a) revealed Na‐Chitosan/MoS_2_/PANI/NF exhibited nearly identical characteristic spectra before and after EA, suggesting that the EA process does not affect the structure of exfoliated MoS_2_ nanosheets and stabilizes their presence in the PANI matrix. This result also suggests that the presence of Na‐Chitosan is crucial for stabilizing the MoS_2_ structure during EA and acts as a bridge connecting MoS_2_ with PANI. In addition, XPS analysis also demonstrated that EA treatment promotes the interaction between MoS_2_ and PANI. The full spectrum is shown in Figure S24 (Supporting Information). In the original PANI spectrum shown in Figure S25 (Supporting Information), characteristic peaks of C─C/C═C, C─N/C═N, and C─N^+^/C═N^+^ were observed at 284.2, 284.9, and 285.8 eV, respectively. After EP, these characteristic peaks shifted toward higher binding energies in the Na‐Chitosan/MoS_2_/PANI/NF spectrum, indicating mutual repulsion between MoS_2_ and PANI in the EP environment. This leads to the tendency of PANI to self‐aggregate, resulting in shifts of its characteristic peaks toward higher binding energies. Interestingly, after EA treatment, the characteristic peaks of C─N/C═N and C─N^+^/C═N^+^ returned to their original positions, compared to the original PANI, while the C─C/C═C peak shifted to a lower binding energy at 283.8 eV. This demonstrates specific interactions occur between the polymer backbone of PANI and the active atomic sites on the surface of MoS_2_,^[^ ^25^, ^35^ ^]^ further implying the construction of P‐N heterojunction interfaces through this non‐covalent bonding pathway. In other words, EA treatment promotes the interaction between MoS_2_ and PANI and leads to the formation of P‐N heterojunction interfaces, which are essential for the efficient electrochemical catalytic system for HER.

To further understand why the Na‐Chitosan/MoS_2_/PANI/NF electrode exhibits unique and efficient HER catalytic ability after EA treatment, we evaluated electrochemical activity surface area (EASA) by measuring the electrochemical double‐layer capacitances (*C*_dl_).^[^ ^52^ ^]^ As shown in Figure 5b and Figure S26 (Supporting Information), the EASA of Na‐Chitosan/MoS_2_/PANI/NF electrodes before and after EA treatment were 13.1 and 2.6 mF cm^−2^, respectively, indicating that Na‐Chitosan/MoS_2_/PANI/NF had a larger surface roughness after EA treatment, which facilitates the generation of numerous reaction catalytic sites and thereby significantly enhances the overall HER efficiency. Moreover, the EA‐treated Na‐Chitosan/MoS_2_/PANI/NF electrode maintained the same LSV curve and Tafel slope values, even after 24 h of potentiostatic testing at a constant current of 100 mA (Figure 5c,d; Figure S27, Supporting Information). Even under high current conditions of 500 mA, the electrode exhibited low overpotential (22.1 mV at −10 mA cm^−2^) and an acceptable Tafel slope value after 24 h, indicating excellent long‐term structural stability and durability. Notably, after 24 h under high current conditions of 500 mA, the region covered by Na‐Chitosan/MoS_2_/PANI on the NF substrate maintained its structural integrity, whereas the free NF region exhibited significant damage (Figure S28, Supporting Information). This further confirms that the EP and EA processes effectively and uniformly deposit Na‐Chitosan/MoS_2_/PANI on the NF substrate, thereby improving the environmental resistance or tolerance of the NF substrate. Collectively, this newly proposed method for preparing exfoliated MoS_2_ nanosheet‐based catalysts with P‐N heterojunction interfaces not only exhibits excellent HER performance, low overpotential, and structural stability, but also demonstrates promising potential for future development compared to Pt/C catalysts.

**Figure 6:**
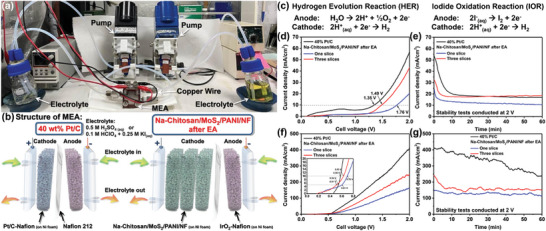
a) Photograph of the PEMFC workstation used in this study. b) Structure of the MEA: on the left is the control group with the Pt/C‐Nafion system; on the right is the main system with one or three slices of Na‐Chitosan/MoS_2_/PANI/NF electrodes after EA treatment. c) Reaction equations for the HER and IOR in an acidic aqueous solution. Current density–voltage curves of the PEMFCs in d) 0.5 m H_2_SO_4_ solution at 25 °C and f) 0.1 M HClO_4_/0.25 m KI solution at 40 °C. Current density response of PEMFCs during continuous operation at 2 V for 60 min in e) 0.5 m H_2_SO_4_ solution at 25 °C and in g) 0.1 m HClO_4_/0.25 m KI solution at 40 °C. The inset in f) presents an enlarged view of the low‐voltage and low‐current density region.

Proton exchange membrane fuel cells (PEMFCs) are an alternative energy technology that generates electricity through the reaction between hydrogen (or hydrogen‐rich derivatives) and oxygen. PEMFCs have garnered significant attention from academia and industry due to their high energy conversion efficiency, power density, low cost, and operating temperature.^[^ ^53^ ^]^ Membrane electrode assembly (MEA) is a critical component that significantly impacts the performance of PEMFCs, as the MEA facilitates the electrochemical reactions required for the separation and recombination of protons and electrons.^[^ ^54^ ^]^ Therefore, we conducted preliminary evaluations of the overall device efficiency using a PEMFC assembly with Na‐Chitosan/MoS_2_/PANI/NF electrodes after EA treatment serving as the cathode catalyst. The PEMFC testing equipment is illustrated in **Figure** **6**a. The cathode and anode within the MEA each have a space size of 2 cm × 2 cm × 0.5 cm (length × width × height). The internal structure of the MEA is illustrated in Figure 6b. The anode catalyst was the noble metal catalyst iridium dioxide (IrO_2_) loaded on NF; the cathode catalyst was either Pt/C (Pt 40 wt.%) or Na‐Chitosan/MoS_2_/PANI/NF after EA treatment; the proton exchange membrane was composed of Nafion 212 (with a thickness of 0.05 mm); the electrolyte was either 0.5 m H_2_SO_4_ solution for the HER or a mixture of 0.1 m perchloric acid (HClO_4_) and 0.25 m potassium iodide (KI) for the iodide oxidation reaction (IOR). The evaluations of the HER and IOR were conducted at 25 and 40 °C, respectively, with the cathode and anode reactions depicted in Figure 6c. A systematic investigation was conducted to directly confirm the overall performance of the electrode to produce hydrogen and oxygen in the HER, and also explore the reduction reaction of iodine ions at the anode and the hydrogen production efficiency at the cathode in the IOR.^[^ ^55^ ^]^ Moreover, the results of IOR can further validate the results of HER.

Considering the content of the MoS_2_ catalyst on the electrode (Figure 4g) and the space at the cathode of the MEA, we investigated the effect of MoS_2_ content on the performance of PEMFC by placing one slice or three slices of the Na‐Chitosan/MoS_2_/PANI/NF electrode after EA treatment at the cathode (Figure 6b). The results for the HER are shown in Figure 6d. Pt/C achieved a turn‐on voltage of 1.35 V at a current density of 10 mA cm^−2^ and reached a maximum current density of 57 mA cm^−2^ at 2 V. In comparison, the turn‐on voltage of the one‐slice Na‐Chitosan/MoS_2_/PANI/NF electrode after EA treatment was 1.76 V and the maximum current density at 2 V was 20 mA cm^−2^, suggesting that the lower performance at operating voltage may be due to the limitation imposed by the content of MoS_2_ on the electrode. Interestingly, when the Na‐Chitosan/MoS_2_/PANI/NF electrode after EA treatment was increased to three slices, the turn‐on voltage significantly decreased to 1.49 V and the maximum current density at 2 V increased to 43 mA cm^−2^. This confirms that increasing the number of slices (i.e., increasing the content of MoS_2_) effectively reduces the energy barrier of the HER, thus improving the efficiency of hydrogen and oxygen production. Thus, controlling the internal space of the MEA and the number of cathode catalyst slices has the potential to achieve higher performance and also provides greater flexibility to optimize the MEA stack system. To confirm the stability and reliability of these MEAs, continuous operation tests were conducted for 60 min at a fixed voltage of 2 V. As shown in Figure 6e, the current density of all MEAs rapidly decreased within the first 10 min and then gradually reached a steady state. These features are commonly observed during constant voltage (or constant current) switch tests of MEAs and can be attributed to two main phenomena occurring under the initial electrolyte conditions upon voltage application: mass transport polarization or changes in the oxidation state of the anode electrode surface.^[^ ^56^ ^]^ After the initial unstable period, the three‐slice Na‐Chitosan/MoS_2_/PANI/NF electrode after EA treatment exhibited a stable output current density, reaching 19 mA cm^−2^ at 60 min, slightly higher than Pt/C at 17 mA cm^−2^, indicating that the electrochemically activated Na‐Chitosan/MoS_2_/PANI/NF electrode holds potential for applications in PEMFCs. The same trend was also observed in the IOR. As shown in Figure 6f, compared to the HER (Figure 6d), the turn‐on voltage of all MEAs at the same current density was significantly lower, highlighting the low‐energy consumption advantages of the IOR for hydrogen and iodine production.^[^ ^55^ ^]^ Notably, at a current density of 5 mA cm^−2^, the turn‐on voltage of the three‐slice Na‐Chitosan/MoS_2_/PANI/NF electrode after EA treatment was 0.51 V, slightly lower than the 0.54 V of Pt/C (inset of Figure 6f), indicating a low energy barrier and high catalytic activity. When operated at 2 V, the Na‐Chitosan/MoS_2_/PANI/NF electrode with three slices achieved a current density of 250 mA cm^−2^, while Pt/C reached 408 mA cm^−2^. When further evaluated at a constant voltage of 2 V for 60 min, the current density of Pt/C gradually decreased over time (Figure 6g). In contrast, after the initial unstable period, the current density of the three‐slice Na‐Chitosan/MoS_2_/PANI/NF electrode after EA treatment remained stable at ≈160 mA cm^−2^ for the entire 60 min, indicating excellent catalytic stability. Based on the HER and IOR results obtained using the PEMFC devices, the Na‐Chitosan/MoS_2_/PANI/NF electrode after EA treatment can be considered an efficient non‐noble metal‐based electrochemical HER catalyst. Due to the presence of P‐N heterojunction interfaces, this novel catalyst not only facilitates efficient hydrogen production at the cathode of MEAs and promotes stable operation of PEMFCs, but also holds the potential to replace commercial Pt/C catalysts and contribute to future developments in the field of energy.

## 3 Conclusion

In summary, we are the first to successfully demonstrate the construction of a Na‐Chitosan/MoS_2_/PANI/NF electrode with P‐N heterojunction interfaces and high catalytic activity through the combination of Na‐Chitosan‐assisted MoS_2_ exfoliation, the EP reaction of PANI, and EA treatment. This electrode exhibits excellent electrochemical catalytic performance and device efficiency as a cathode catalyst in PEMFCs. The discovery of this process could not only enable the development of low‐cost, efficient, controllable, and scalable Na‐Chitosan/MoS_2_/PANI/NF electrodes as non‐noble metal‐based electrochemical catalysts, but also holds great potential to replace commercial Pt/C catalysts and be widely applied in the field of energy. During the preparation of water‐dispersible exfoliated MoS_2_ nanosheets, due to the high affinity between the synthesized Na‐Chitosan and bulk MoS_2_ crystals, a combination of simple ultrasonic treatment and adjustment of the blending ratio of Na‐Chitosan and MoS_2_ can easily control the solid content, long‐term dispersion stability, layer number, and physical properties of the resulting exfoliated MoS_2_ nanosheets in water. In particular, the solid Na‐Chitosan/MoS_2_ composite material can be stably preserved by freeze‐drying and also easily re‐dispersed in water through the simple addition of water and stirring. This further indicates that Na‐Chitosan can be regarded as a unique functional dispersal agent that provides an efficient and reliable approach to constructing water‐dispersible 2D nanocomposites. In addition, due to the P‐type and N‐type semiconductor characteristics of PANI and MoS_2_, respectively, Na‐Chitosan/MoS_2_ nanosheets can be successfully incorporated in the PANI matrix through the EP process on the NF substrate. Moreover, after EA treatment, the stable formation of P‐N heterogeneous interface interfaces between PANI and MoS_2_ ultimately constructs Na‐Chitosan/MoS_2_/PANI/NF electrodes with electrochemical catalytic properties. In the electrochemical evaluation of the HER, compared to the commercial Pt/C catalyst, the Na‐Chitosan/MoS_2_/PANI/NF electrode after EA treatment exhibited significantly lower resistance (1.7 ohms) and overpotential (−42.7 and −76.1 mV at −50 and −500 mA cm^−2^, respectively), a comparable Tafel slope (26.3 mV dec^−1^), and good catalytic stability (operating for 24 h at 100 and 500 mA) in 0.5 m H_2_SO_4_ electrolyte, indicating that this non‐noble metal‐based electrochemical catalytic system may offer excellent HER catalytic performance. More importantly, measurements of the HER and IOR in PEMFC devices confirmed that the Na‐Chitosan/MoS_2_/PANI/NF electrode after EA treatment exhibits a low turn‐on voltage (1.49 V for HER and 0.63 V for IOR at 10 mA cm^−2^), high current density (43 mA cm^−2^ for HER and 250 mA cm^−2^ for IOR at 2 V), and can operate steadily for 60 min at 2 V. Thus, this newly developed system efficiently produces hydrogen at the cathode of MEA and promotes the stable operation of PEMFCs, and also holds potential as an alternative to Pt/C with wide applicability in various energy fields. Collectively, this study provides a low‐cost, facile, and efficient approach for the preparation of water‐dispersible exfoliated 2D nanomaterials, the development of electrochemical catalytic composites with P‐N heterojunction interfaces, and the fabrication of reliable electrochemical catalytic cathodes for PEMFC devices, and therefore holds great potential to make significant contributions to the realization of high‐performance PEMFCs and the development of green energy.

## 4 Experimental Section

The Experimental Section in the Supporting Information contains detailed information on the chemicals, instruments, materials synthesis, analysis and identification, electrochemical, and fuel cell experiments.

## Conflict of Interest

The authors declare no conflict of interest.

## Supporting information

Supporting Information

Supplemental Video 1

## Data Availability

Research data are not shared.
