# Supplementary material for: Exfoliated 2D Nanosheet‐Based Conjugated Polymer Composites with P‐N Heterojunction Interfaces for Highly Efficient Electrocatalytic Hydrogen Evolution
Source: Adv Sci (Weinh). 2024 Jul 31;11(36):2407061. doi: 10.1002/advs.202407061 (PMC11423191; doi:10.1002/advs.202407061)
Supplement: Supplementary file 1 — Supporting Information [file ADVS-11-2407061-s001.docx]

**Supplementary Information**

Exfoliated Two-Dimensional Nanosheet‐Based Conjugated Polymer Composites with P-N Heterojunction Interfaces for Highly Efficient Electrocatalytic Hydrogen Evolution

Cheng-Yu Tsai,^a^ Hsu-Sheng Li,^a^ Kumasser Kusse Kuchayita,^a^ Hsin-Chih Huang,^c^ Wei-Nien Su^a^ and Chih-Chia Cheng^ab^*

1. Graduate Institute of Applied Science and Technology, National Taiwan University of Science and Technology, Taipei 10607, Taiwan.
   E-mail: cccheng@mail.ntust.edu.tw
2. Advanced Membrane Materials Research Center, National Taiwan University of Science and Technology, Taipei 10607, Taiwan.
3. Department of Materials Science and Engineering, National Formosa University, Yunlin 63201, Taiwan.

* Corresponding author is marked with an asterisk (*) in the complete list of authors.

**Experimental section**

**Chemicals and reagents**

All chemicals were of the highest grade available. Chitosan powder [average molecular weight (*M*_w_) of 200,000 g/mol, 85% degree of deacetylation) was obtained from GeneFerm Biotechnology Co., Ltd. (Tainan City, Taiwan). Commercial bulk molybdenum(IV) sulfide (MoS_2_, 99.9%), molybdenum(IV) selenide (MoSe_2_, 99.9%), tungsten(IV) sulfide (WS_2_, ≥99%), aniline (99.5%), sodium carboxymethylcellulose powder (Na-CMC, degree of substitution 0.7, *M*_w_ of 250,000 g/mol) and Nafion solution (5 wt%) were purchased from Sigma-Aldrich (Louis, MO, USA). Sulfuric acid (H_2_SO_4_, 97%) and hydrochloric acid (HCl, 37%) were purchased from Honeywell Fluka (Morristown, NJ, USA) and used without further treatment; 20 wt% or 40 wt% platinum on carbon (20 wt% or 40 wt% Pt/C) and iridium (IV) oxide powder (IrO_2_, 99%) were purchased from Alfa Aesar (Ward Hill, MA, USA). All other reagents and solvents were obtained from Sigma-Aldrich or TEDIA (Fairfield, OH, USA). Nickel foam (NF) with a purity > 99.99%, density of 346 g/m^2^, thickness of 1.6 mm, and porosity > 95% (80-110 pores per inch and average hole diameter ~0.25 mm) was sourced from MTI corporation (Richmond, CA, USA).

**Synthesis of sodium-functionalized chitosan (Na-Chitosan)**

Chitosan (2.5 g) and isopropanol (30 mL) were stirred in a 500-mL round-bottomed flask at room temperature for 1 h, then 40% aqueous sodium hydroxide (NaOH, 10 mL) was added and the mixture was stirred continuously for 12 h. Subsequently, methylchloroacetate (10 mL) and isopropyl alcohol (10 mL) were added to the alkaline chitosan solution and stirred at 65 °C under nitrogen gas for 4 h. The resulting suspended solid was collected by vacuum filtration, dried in a vacuum oven for 24 h at 40 °C, transferred to a 250 mL flask and dispersed in aqueous NaOH (40%, 80 mL), followed by stirring at room temperature for 18 h, affording a light-yellow colloidal solution. The sample was diluted in approximately 150 mL of deionized water, dialyzed to a clear solution, and freeze-dried to produce white sponge-like Na-Chitosan (1.5 g, yield: 65%). The chemical structure of Na-Chitosan is presented in **Scheme 1a**.

**Preparation of MoS_2_ dispersion in aqueous solution**

Few-layer MoS_2_ nanosheets were synthesized by a simple liquid phase exfoliation method. Briefly, 1.0 mg/mL Na-Chitosan aqueous solution was prepared by dissolving the functionalized polymer salt in deionized water. Aqueous solutions of Na-Chitosan (1.0 mg/mL) and bulk MS_2_ powder (1 mg, 3 mg, or 5 mg, corresponding to Na-Chitosan/MoS_2_ weight ratios of 1:1, 1:3, and 1:5, respectively) were ultrasonicated for 30 min at 50% amplitude using a probe ultrasonicator (Qsonica, model Q700, Newtown, CT, USA); the samples were kept in an ice bath to maintain the temperature below 5 °C. Subsequently, the dark gray dispersion obtained was filtered and centrifuged for 10 min at 4000 rpm to separate the bulk materials that had not been exfoliated. Using a pipette, the top 85% of the supernatant solution was collected and subjected to characterization and electrocatalyst development. The residual precipitate was dried in a vacuum oven and the amount of MS_2_ exfoliated from the pristine bulk material was estimated based on the difference between initial and residual weights.

For the MoSe_2_ and WS_2_ systems, the optimal concentration of Na-Chitosan for exfoliation and dispersion of MoSe_2_ or WS_2_ was determined by dispersing bulk MoS_2_ in various concentrations of aqueous Na-Chitosan solutions. After obtaining the dispersed solutions of MoSe_2_ or WS_2_, the effects of Na-Chitosan on exfoliation of MoSe_2_ or WS_2_ crystals were assessed by measuring particle size distribution, surface potential, SEM, and redispersibility after freeze drying.

**Comparison of Na-Chitosan/MoS_2_ composites before and after freeze-drying**

We characterized and evaluated the properties of the nanosheets before and after freeze drying. The dispersion of exfoliated Na-Chitosan/MoS_2_ was freeze dried; the lyophilized samples resembled a sponge with a fluffy porous structure. The freeze-dried samples were dissolved in deionized water (1 mg/mL) and compared to the samples before freeze drying.

**Preparation of anilinium chloride**

In a 20-mL vial, aniline (5.46 mL) was vigorously stirred at room temperature while adding concentrated hydrochloric acid (12.0 mL) in four portions, 3 mL at a time. After continual stirring, the resultant dark brown solution gradually formed pink-purple anilinium chloride crystals. A saturated aqueous solution of anilinium chloride was prepared by dissolving the corresponding salt in deionized water, which was used as a monomer for the electropolymerization (EP) of aniline.

**Electrodeposition of Na-Chitosan/MoS_2_/PANI on NF (Na-Chitosan/MoS_2_/PANI/NF)**

The designed electrodes, comprising MoS_2_ nanosheets grown directly on NF were obtained using a facile EP process using a SP-200 potentiostat workstation (Bio-Logic, Knoxville, TN, USA). The EP process in this work was performed in a three-electrode conﬁguration using NF (2 cm × 2 cm) as the working electrode, silver/silver chloride (Ag/AgCl) as the reference electrode, and platinum wire as the counter electrode. In brief, the three-electrode system was carefully immersed in the growth solution composed of exfoliated Na-Chitosan/MoS_2_ nanosheets and saturated aqueous of anilinium chloride mixed in a 1:2 ratio. EP was performed at an applied chronopotentiometric fixed current of 0.10 mA for different deposition times from 40 to 90 min, and the deposited samples were denoted as Na-Chitosan/MoS_2_/PANI/NF. Finally, the prepared Na-Chitosan/MoS_2_/PANI/NF electrodes were cleaned with drops of deionized water to remove the loosely adhered nanosheets and aniline salt on the surface of nickel foam and air dried naturally at room temperature. A similar procedure was used to electrodeposit PANI into NF for comparison, using saturated aqueous anilinium chloride as the developing solution without exfoliated nanomaterials; the resulting electrode was assigned as PANI/NF. EP of aniline monomers and the deposition of nanosheets on the surface of NF led to deposition of a greenish color on the bare NF substrate, which was further confirmed by the decrease in potentiometric curves. To prepare electrodes with different geometric sizes, we attempted to electrodeposit the active material over deposition areas of 1 cm × 1 cm, 2 cm × 2 cm, and 5 cm × 5 cm, which are suitable for utilization in fuel cells of different sizes.

**Electrochemical activation (EA) of Na-Chitosan/MoS_2_/PANI/NF electrodes**

Na-Chitosan/MoS_2_/PANI/NF electrodes in 0.5 M H_2_SO_4_ electrolyte solution were electrochemically activated via chronoamperometry at a fixed current of 500 mA for 4 h, then rinsed with deionized water several times.

**Characterization**

**Fourier transform infrared (FT-IR) spectra** of chitosan and Na-Chitosan were obtained using a PerkinElmer Spectrum Two IR spectrometer (Buckinghamshire, UK) over the scan range between 500-4000 cm^−1^ and a resolution of 2.0 cm^−1^ using potassium bromide (KBr) discs for the samples.

***Proton and carbon-13 nuclear magnetic resonance (^1^H- and ^13^C-NMR) spectra*** of chitosan and Na-Chitosan were recorded using a Bruker AVIII instrument (Billerica, MA, USA) at 500 MHz to confirm the structure of functionalized chitosan. The chitosan sample was dissolved in deuterium oxide (D_2_O) with 3% deuterated acetic acid (CD_3_CO_2_D) and Na-Chitosan was dissolved in D_2_O, and the samples were transferred to NMR tubes for analysis.

***Gel permeation chromatography (GPC)*** was conducted using a water-based Water Breeze GPC detector with a differential refractive index RI 2414. The retention distribution of Na-Chitosan was established at a flow rate of 1.0 mL/min and a temperature of 40 °C. Subsequently, the Waters Breeze software was used to calculate average weight molecular weight (*M*_w_), number-average molecular weight (*M*_n_), and the polydispersity index (PDI).

***Critical micelle concentration (CMC)*** values were measured using pyrene as a hydrophobic fluorescence probe on a fluorescence spectrophotometer (Hitachi F4500, Tokyo, Japan), as previously described in detail.^[21]^

***Ultraviolet-visible (UV-Vis) spectroscopy*** was performed to determine the absorption and transmission of exfoliated MoS_2_ nanosheets, as well as their dispersion stability over one month, using a UV-Vis spectrophotometer (Jasco V-730, Tokyo, Japan).

***UV-Vis-near-infrared (UV-Vis-NIR) spectra*** were recorded using a UV-Vis-NIR spectrophotometer equipped with an integrating sphere (Hitachi U-4100, Tokyo, Japan), covering the spectral range from 200 to 800 nm. Subsequently, the bandgap energy of the samples was determined using Tauc plot analysis based on the UV-Vis absorption spectra, as previously described.^[48]^ Finally, the band gap (*E*_g_) was calculated based on the UV-Vis-NIR spectra by selecting the onset wavelength corresponding to the longest absorption peak, using the formula:

*E*_g_ = 1240 / (λ_onset_)

**Cyclic voltammetry (CV)** was used to determine the HOMO energy levels on a CHI 600E (CHI Instruments Inc, USA), following a previously outlined procedure.^[49]^ Sample solutions were slowly dripped onto carbon paper and dried at room temperature. Aqueous 0.1 M potassium chloride (KCl) was used as the electrolyte and Ag/AgCl (filled with 3 M KCl) was used as the reference electrode. A platinum wire electrode was used for scanning at a CV scan rate of 50 mV/sec to obtain curves with oxidation-reduction peaks. The HOMO potential was calculated using the iron/iron ion (Fe/Fe^2+/3+^) standard oxidation-reduction potential, with the formula:

HOMO = *E*_ox_ + 4.4 eV

where *E*_ox_ is the oxidation peak potential and the standard potential of Fe/Fe^2+/3+^ was taken as 4.4 eV.

Finally, the LUMO energy levels were calculated using the *E*_g_ and HOMO values obtained by UV-Vis-NIR and CV, respectively, with the following formula:

LUMO = HOMO + *E*_g_

***Dynamic light scattering (DLS) and zeta potentials*** used to assess size distribution and surface charge in water at 25 °C using a Nano Brook Zeta PALS (Brookhaven Instruments Corporation, Holtsville, NY, USA).

***Raman spectroscopy*** were recorded using a Raman spectrometer (Jasco NRS-5100, Tokyo, Japan) within the range of 200-500 cm^-1^ at a resolution of 1.09 cm^−1^ using a 20× objective at 25 °C. After exfoliation and centrifugation, the supernatant dispersions were spin-coated onto the surface of clean silicon wafers at 1500 rpm for 10 s and dried for 24 h in a vacuum oven at 25 °C. A 532 nm He-Ne laser was used as the excitation source. The NFs containing the active nanomaterials were cut into small pieces, dried in a vacuum oven, and used directly for Raman spectroscopy.

***Small-angle and wide-angle X-ray scattering (SAXS and WAXS)*** of freeze-dried samples and bulk MoS_2_ were acquired using the Taiwan Photon Source (TPS) beamline BL13A instrument of the National Synchrotron Radiation Research Center (NSRRC, Taiwan). The dried samples were sealed in polyimide film (Kapton, 12-μm-thick) and scanned at 25 °C using a beam diameter of 1.0 mm and X-ray photons with a wavelength of 0.8267 Å.

***X-ray photoelectron spectroscopy (XPS)*** data were collected using a PHI 5000 Versa Probe III using an Al K α source (ULVAC-PHI. Inc., Chigasaki-shi, Japan). All binding energies were corrected using the binding energy of the adventitious C1s peak at 284.8 eV as an internal standard. The resulting XPS spectra were processed using the XPS peak fitting program of XPS PEAK 4.1 software to obtain a linear fitting background and separate the peaks. High-resolution spectra were acquired at a constant pass energy of 10 eV and an energy step of 0.1 eV.

***Atomic force microscopy (AFM)*** samples were spin-coated onto the surface of silicon substrates at 1500 rpm for 10 s and dried in a vacuum oven for 24 h at room temperature. AFM images of exfoliated MoS_2_ nanosheets and bulk MoS_2_ were evaluated using a tapping-mode AFM (NX10, AFM Park Systems, Suwon, South Korea) at 25 °C. The layer numbers were estimated from the thickness data obtained using Park XEI image processing software.

***Scanning electron microscopy (SEM)*** samples were prepared by spin coating one drop of sample solution onto the surface of silicon wafer substrates at 1500 rpm for 10 s, dried in a vacuum oven for 24 h at 25 °C to remove water, and then coated with platinum. The morphology of the nanosheets was assessed by high-resolution field emission SEM (JSM-6500F, JEOL, Tokyo, Japan). Elemental distribution and composition were examined by energy-dispersive X-ray spectroscopy (EDS, Thermo Scientific Ultra Dry SDD EDS, dual detector with software NSS at 15.0 kV accelerating voltage using an aluminium sample holder) and the CHNS-Elemental Analyzer (Thermo Flash 2000 using a tin sample holder).

***High-Resolution Transmission Electron microscopy (HRTEM)*** was used to evaluate the microstructure and morphology of exfoliated MoS_2_ and NF composites on a H-7000 (Hitachi, Tokyo, Japan) operating at 200 kV. The samples for TEM measurements were prepared by drop-casting the sample solution onto a carbon-coated copper grid with a micropipette, followed by drying in a vacuum oven at room temperature for 24 h to remove the solvent. Samples deposited on a nickel foam substrate were embedded in resin, dried in a vacuum oven at room temperature, sectioned using an cryo-ultramicrotome (Leica EM UC6, Germany), and attached to a carbon-plated copper grid. The elemental composition and distributions of the samples were evaluated via EDS and elemental mapping.

**Electrochemical measurements**

The electrocatalytic HER activity of Na-Chitosan/MoS_2_ composites was assessed by depositing the nanosheets and PANI onto the surface of NF via the EP and EA methods to create working electrodes. All electrochemical measurements were performed in a three-electrode configuration using a potentiostat (BioLogic, SP-200, France) at room temperature in 0.5 M H_2_SO_4_ electrolyte solution, using platinum wire as the counter electrode, Ag/AgCl as the reference electrode, and NF (2 cm × 2 cm) as the working electrode.

***Linear sweep voltammetry (LSV)*** was performed at a 10 mV/s scan rate. Before the HER measurements, the electrodes were pre-treated by cycling the potential between -0.8 and +0.2 V vs. reference electrode at a sweep rate of 100 mV/s for 30 cycles to activate the catalysts, remove surface contamination, and stabilize electrochemical current.

***Electrochemical impedance spectroscopy (EIS)*** was performed in the frequency range of 10^5^ to 0.1 Hz at an open-circuit potential with a modulation amplitude of 10 mV, and a Randles equivalent circuit model was used to fit the EIS Nyquist plots. All the potentials reported in this work were converted into the potential of a reversible hydrogen electrode (RHE) scale using the Nernst equation (*E*_RHE_ =  *E*_Ag/AgCl_  +  0.059 pH + *E*^°^_Ag/AgCl_); where *E*^°^_Ag/AgCl_ = 0.21 V at 25 °C and *E*_Ag/AgCl_ is the experimental measured potential. All polarization curves were subjected to *iR* correction, where *i* denotes current and *R* denotes series resistance resulting from the resistances of the substrate and electrolyte.

Surface characterization of the developed electrodes

After EP and EA of the electrocatalysts developed on the surface of NF, the samples were cut into smaller slices for surface characterization using XPS, SEM, HRTEM and Raman spectroscopic methods to confirm the morphological changes on the surface of NF before and after electrode reactivation.

**Stability testing of the developed electrodes**

Chronoamperometry was performed in 0.5 M H_2_SO_4_ electrolyte solution and a fixed current density (100 mA or 500 mA) to record the variation of the potential over time (V-t curves) for Na-Chitosan/MoS_2_/PANI/NF electrodes after EA treatment.

**Parameters used to evaluate catalytic activity**

The catalytic activity of catalysts was evaluated and compared using the following parameters:

***Overpotential (η)***: An extra potential (overpotential; symbolized as η) is required to overcome the intrinsic kinetic hindrance in electrochemical water splitting in both the HER and oxygen evolution reaction (OER). The reversible thermodynamic potential for the HER and OER are 0 and 1.23 V vs. RHE. The overpotential values for each experiment were calculated using the following relation, where '*E*_obs_' implies iR-corrected potential value vs. RHE), *η* (HER) = (0 - *E*_obs_) V vs*.* RHE. The overpotential at a current density of -10 mA/cm^2^ abbreviated as *η*_10_ is a benchmark for electrocatalytic HER/OER.

***Tafel slope*** was calculated by fitting the overpotential vs. log/(j)/ using the Tafel equation as given below (by assuming identical concentration in bulk and at the electrode-electrolyte interface), $=blog(\frac{j}{j_{0}})$, where 'b' implies the Tafel slope value, '*j*' implies the current density value, and '*j*_0_' is the exchange current density.

***Electrochemical active surface area (EASA)*** was measured by determining the electrochemical double layer capacitance (*C*_dl_) using $\text{E}\text{A}\text{SA}\text{= }\frac{\text{C}_{\text{dl}}}{\text{C}_{\text{s}}}$, where 'C_s_' denotes a specific capacitance value of 0.040 mF/cm^2^ depending on the typical reported values.

**HER and IOR testing in membrane electrode assemblies (MEAs)**

***Preparation of Pt/C on NF:*** Homogenous Pt/C catalyst ink was prepared using a standard procedure, as follows: 3.0 mg of commercial 40% Pt/C catalyst powder was dispersed into a 450-μL mixture of water, isopropanol (2:3), and 50-μL Nafion (5 wt%), then the mixture was sonicated for 1.5 h in a water bath sonicator. Subsequently, the Pt/C-Nafion/NF electrode was formed by painting the surface of NF (2 cm × 2 cm) with the as-prepared catalyst ink and the ink was allowed to dry naturally.

***Preparation of IrO_2_ on NF:*** The anodic IrO_2_ catalyst was prepared using a similar procedure as the Pt/C-Nafion/NF electrode. A solution of IrO_2_ (2 mg) mixed with Nafion (50 μL) in water was coated onto NF (2 cm × 2 cm) to construct the IrO_2_-Nafion/NF electrode.

***MEAs for HER and IOR testing***: The anode catalyst was IrO_2_-Nafion/NF, while the cathode was the developed Na-Chitosan/MoS_2_/PANI/NF electrodes after EA treatment. The proton exchange membrane fuel cell (PEMFC) workstation and the structure of the MEAs are shown in **Figures 6a** and **6b**, respectively. The MEAs were fabricated using a Nafion-212 membrane as a polymer electrolyte membrane sandwiched between the two catalyst electrodes. The Nafion 212 membranes were cut into 2.25 × 2.25 cm^2^ pieces (with a thickness of 0.05 mm) and cleaned by soaking for 1 h at 80 °C in 3% H_2_O_2_, 0.5 M H_2_SO_4_, and deionized water. Finally, the MEAs were made both with and without hot-pressing techniques. For the activated electrocatalysts, the active electrode areas of the cells were 4.0 cm^2^ (2 cm × 2 cm) and 5.0625 cm^2^ (2.25 cm × 2.25 cm), respectively. The HER tests were performed with 0.5 M H_2_SO_4_ aqueous solution at 25 °C at one atmospheric pressure. The IOR tests were conducted with 0.1 M HClO_4_ and 0.25 M KI at 40 °C at one atmospheric pressure. Prior to recording the polarization curve, the electrolyte was circulated in a closed circuit at the anode and negative ends of the MEA using a pump at a flow rate of 100 mL/min for 5 min. The performance of MEAs was evaluated by recording the polarization curves from 0 to 2 V at a scan rate of 10 mV/s. The durability of the MEAs was assessed at a fixed applied voltage of 2 V for 60 min.

**Statistical analysis**

All electrochemical analyses and MEA experiments were evaluated through at least three independent experiments to confirm the reproducibility of the results.


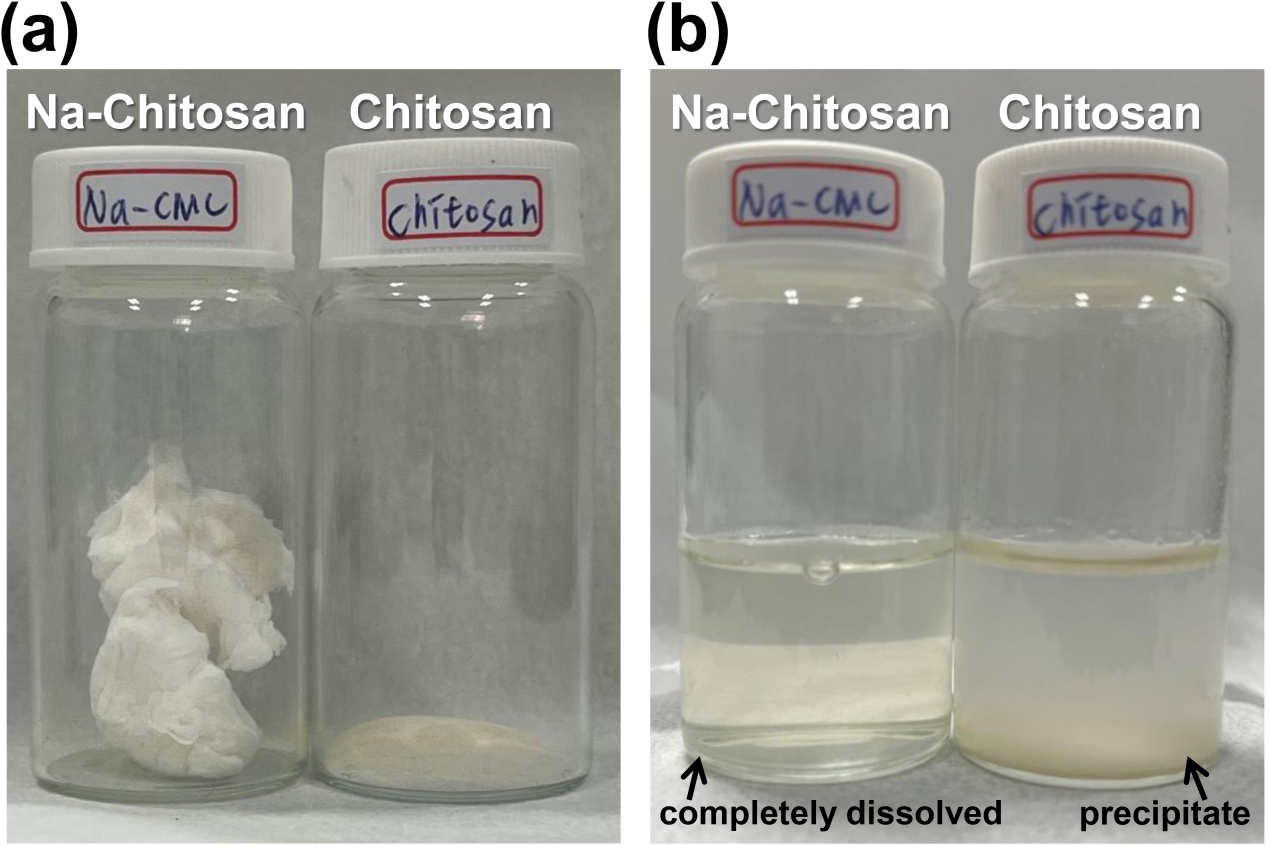


**Figure S1:** Na-Chitosan and Chitosan in **(a)** the solid state and **(b)** in aqueous solution at 25 °C.

**
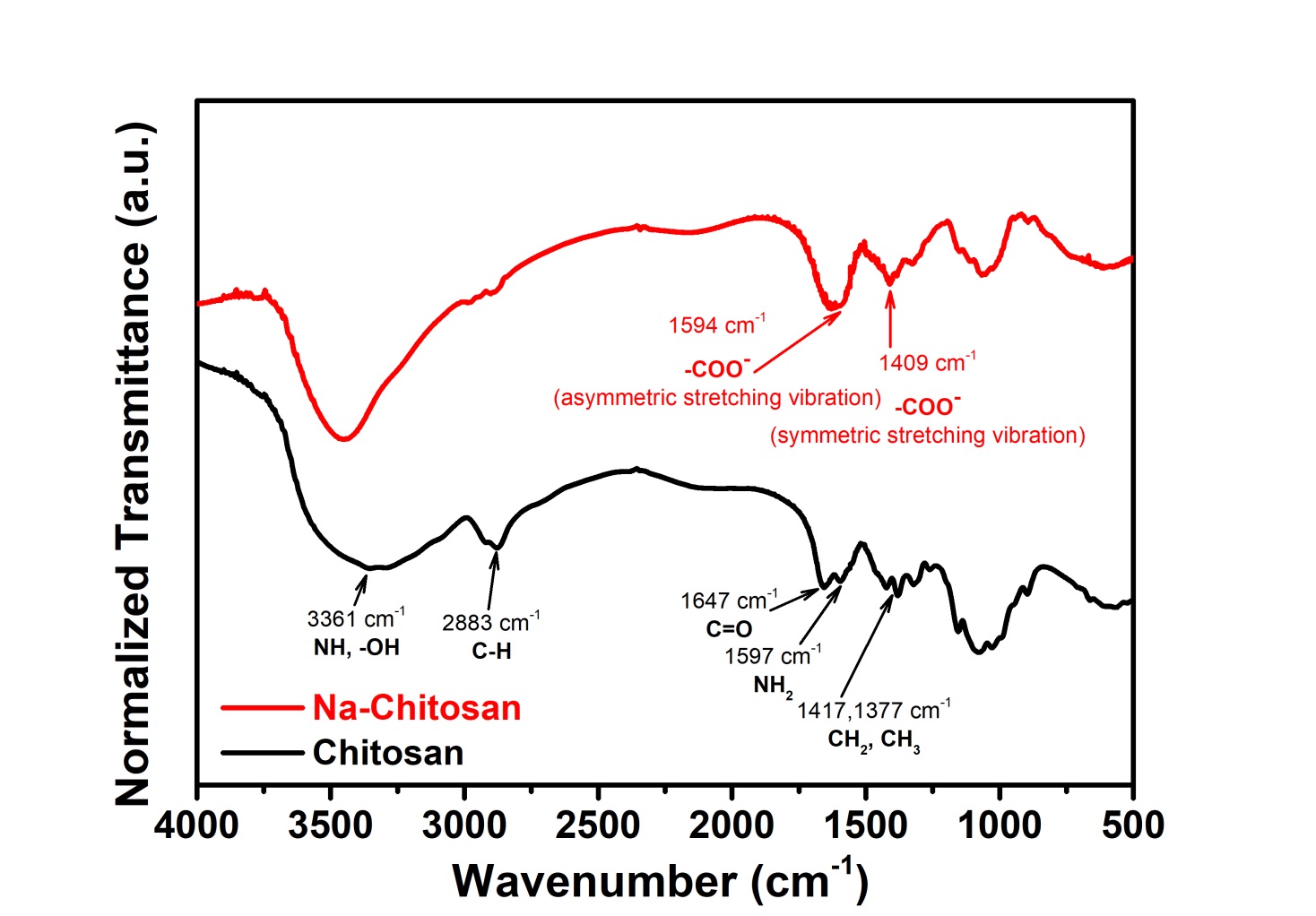
**

**Figure S2:** FTIR spectra of Na-Chitosan and Chitosan obtained at 25 °C.

**
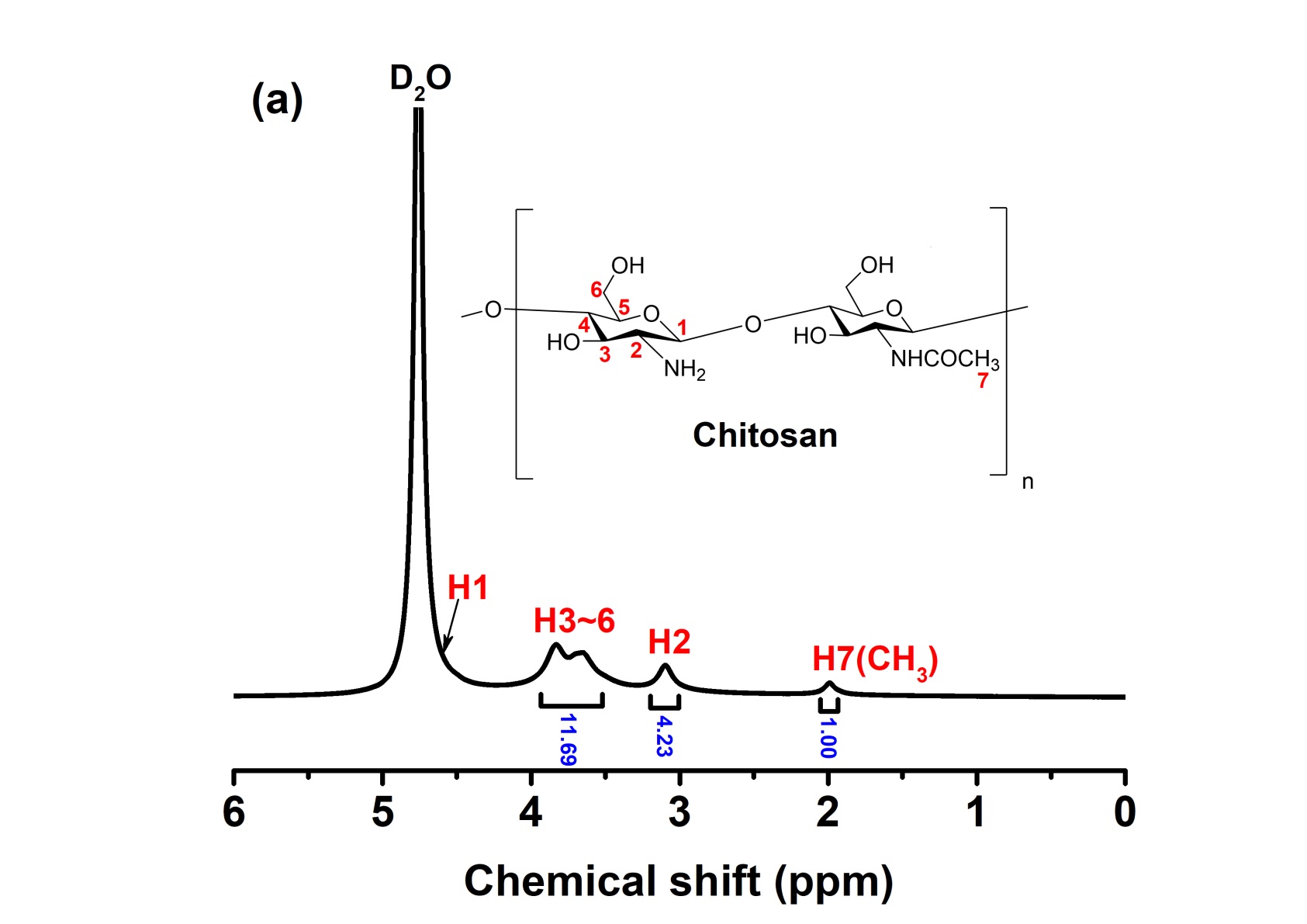
**

**
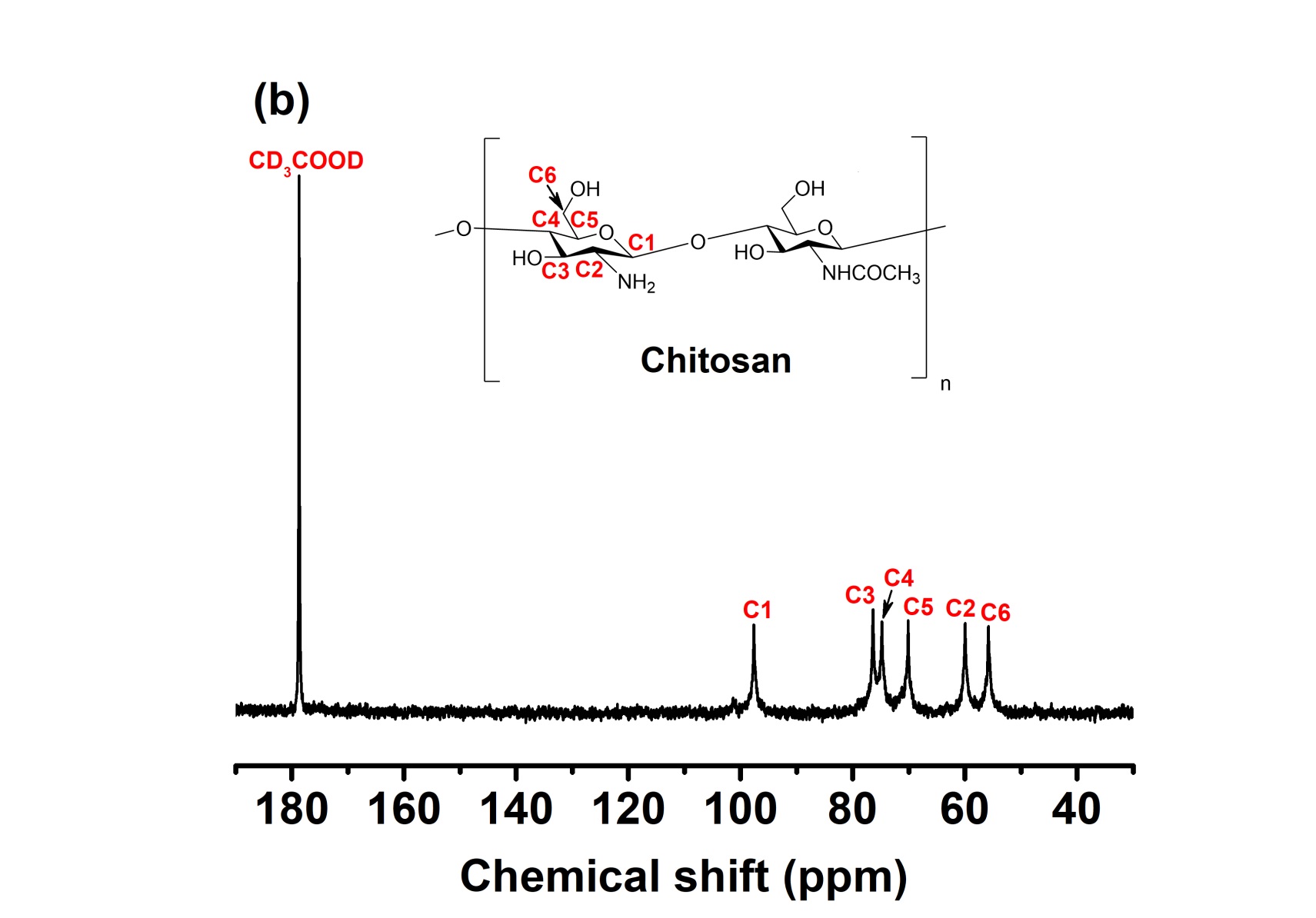
**

**Figure S3: (a)** ^1^H and **(b)** ^13^C NMR spectra of Chitosan in deuterium oxide (D_2_O) with 3% deuterated acetic acid (CD_3_CO_2_D) at 20 °C.

**
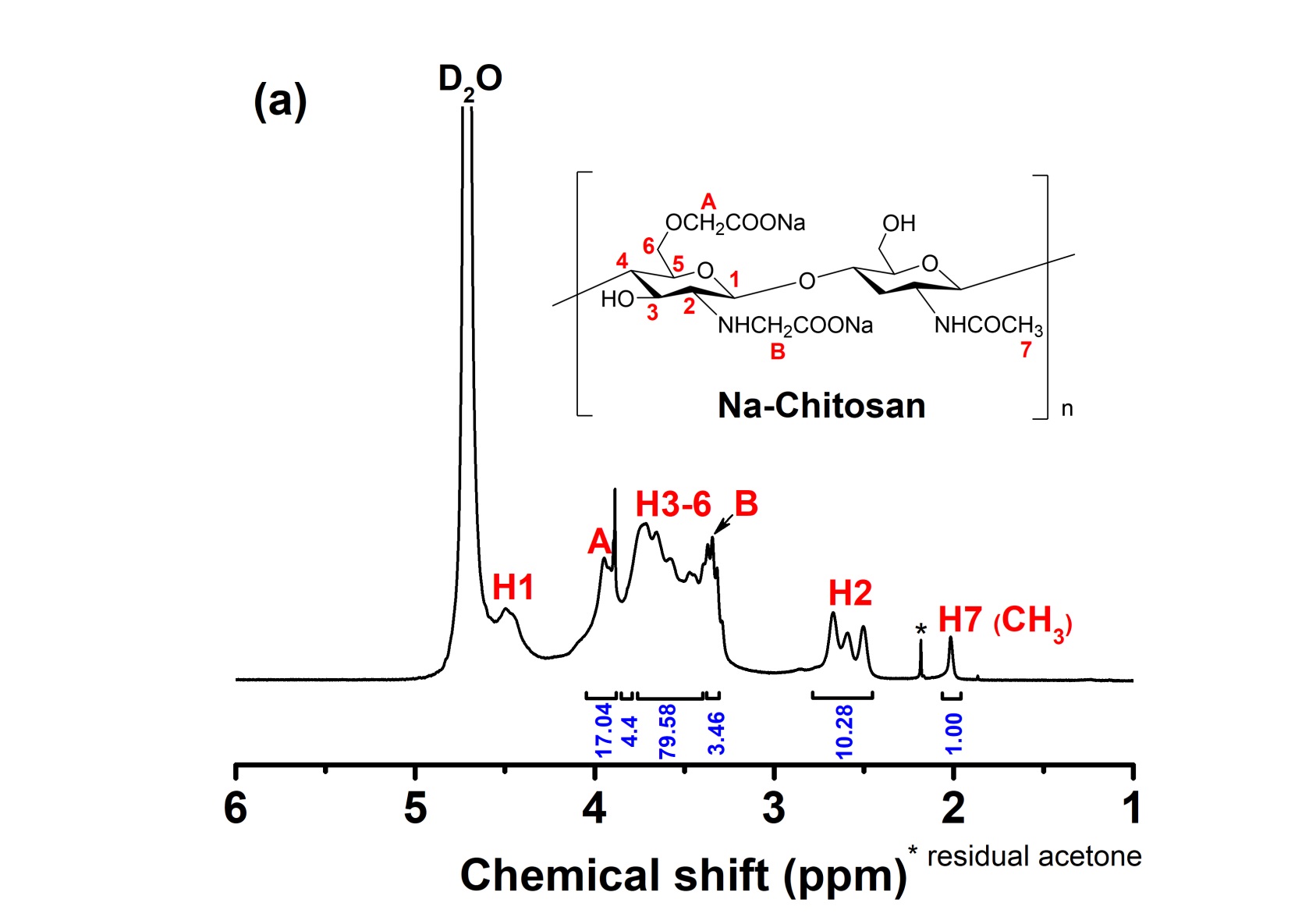
**

**
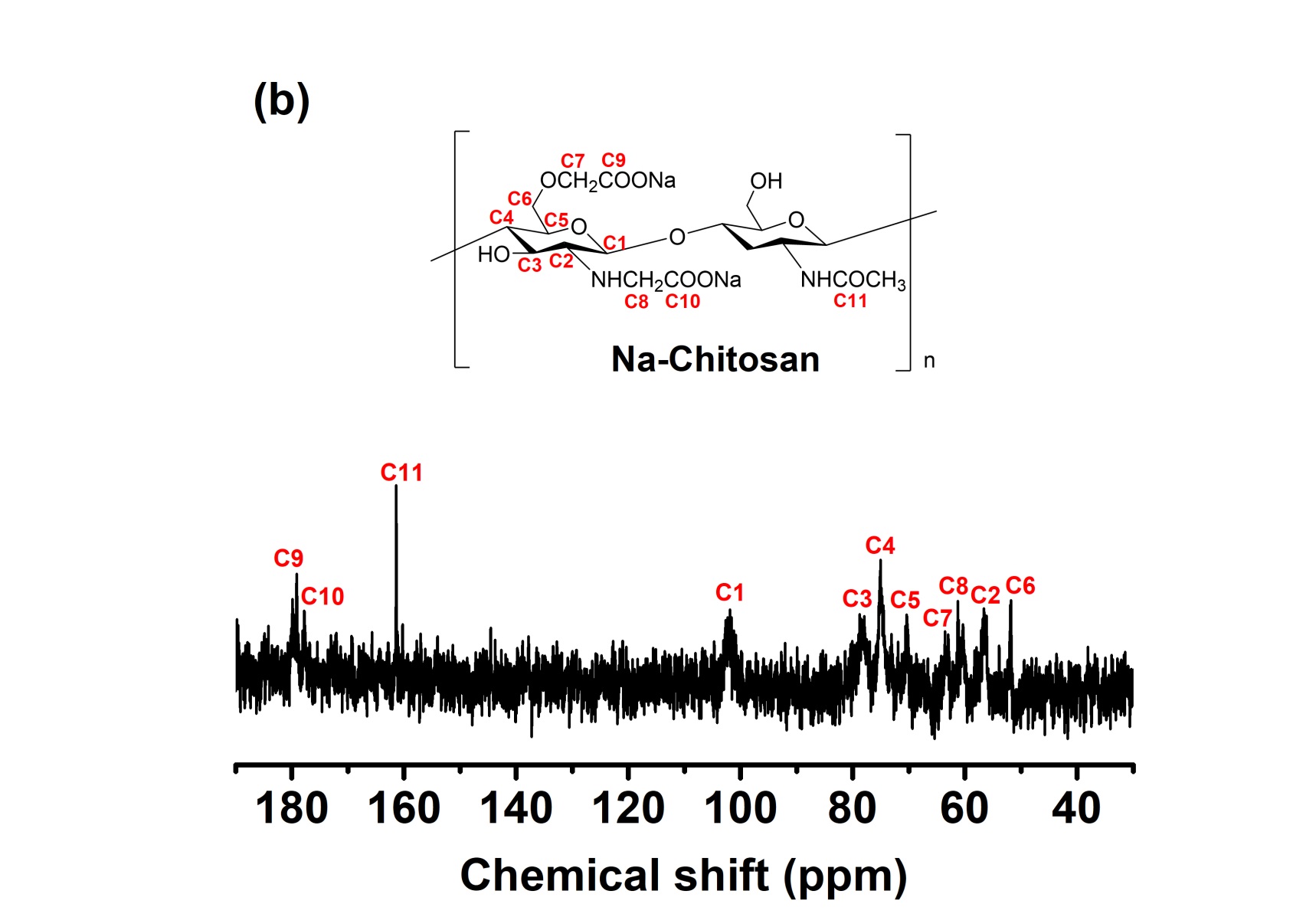
**

**Figure S4: (a)** ^1^H and **(b)** ^13^C NMR spectra of Na-Chitosan in D_2_O at 20 °C.

**
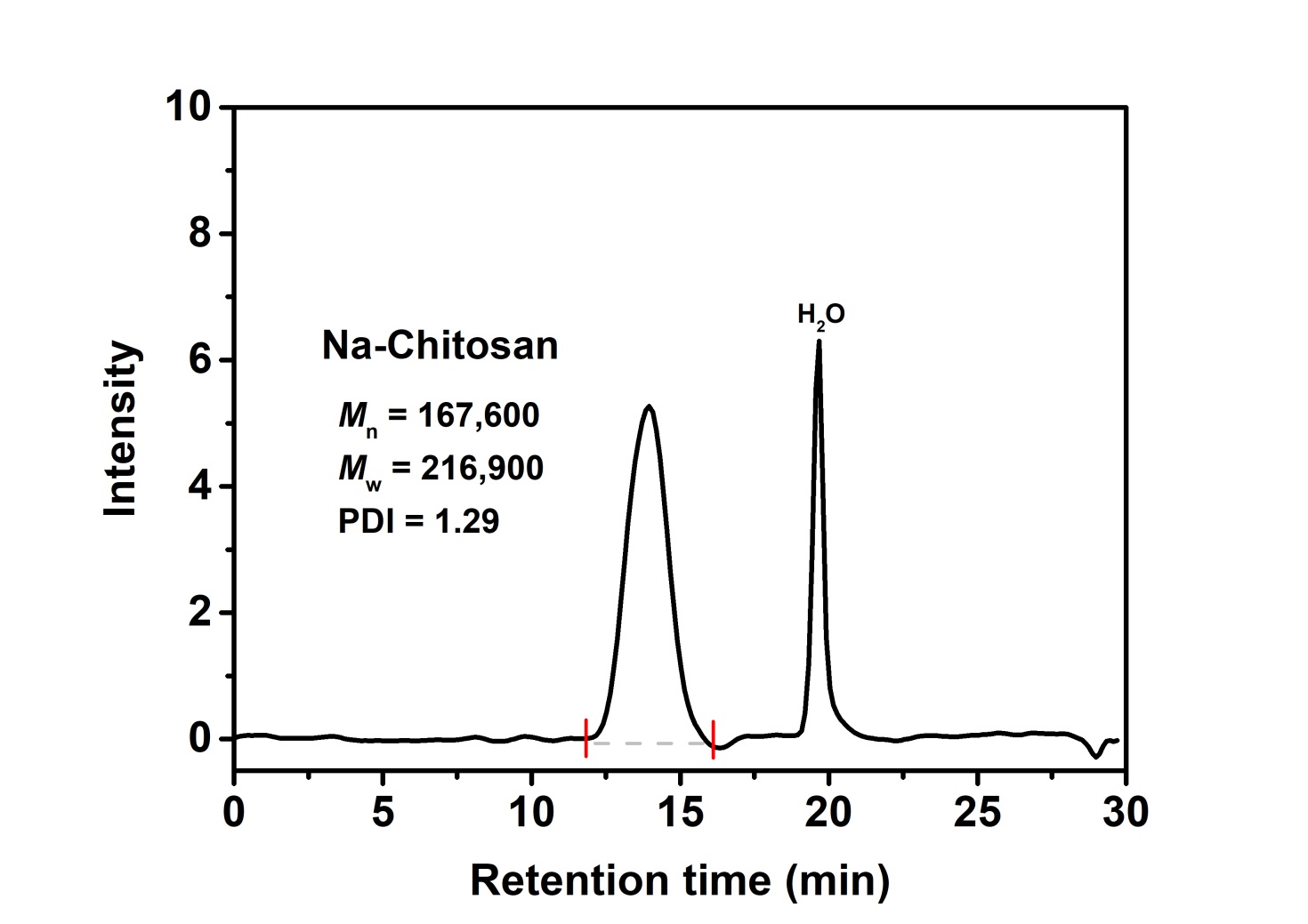
**

**Figure S5:** Water-based GPC trace of Na-Chitosan and its molecular weight information.

**
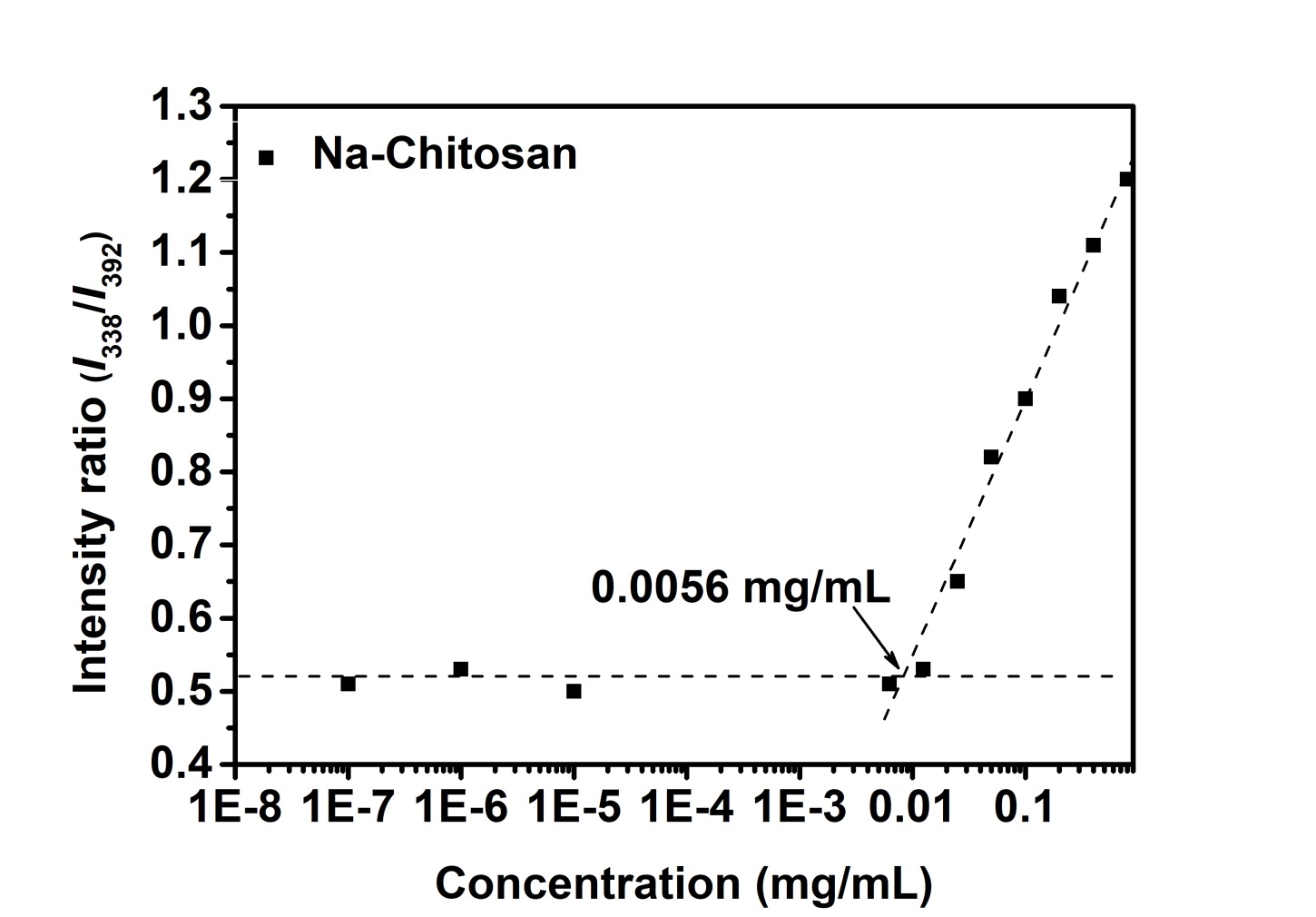
**

**Figure S6:** CMC curve of Na-Chitosan in aqueous solution.


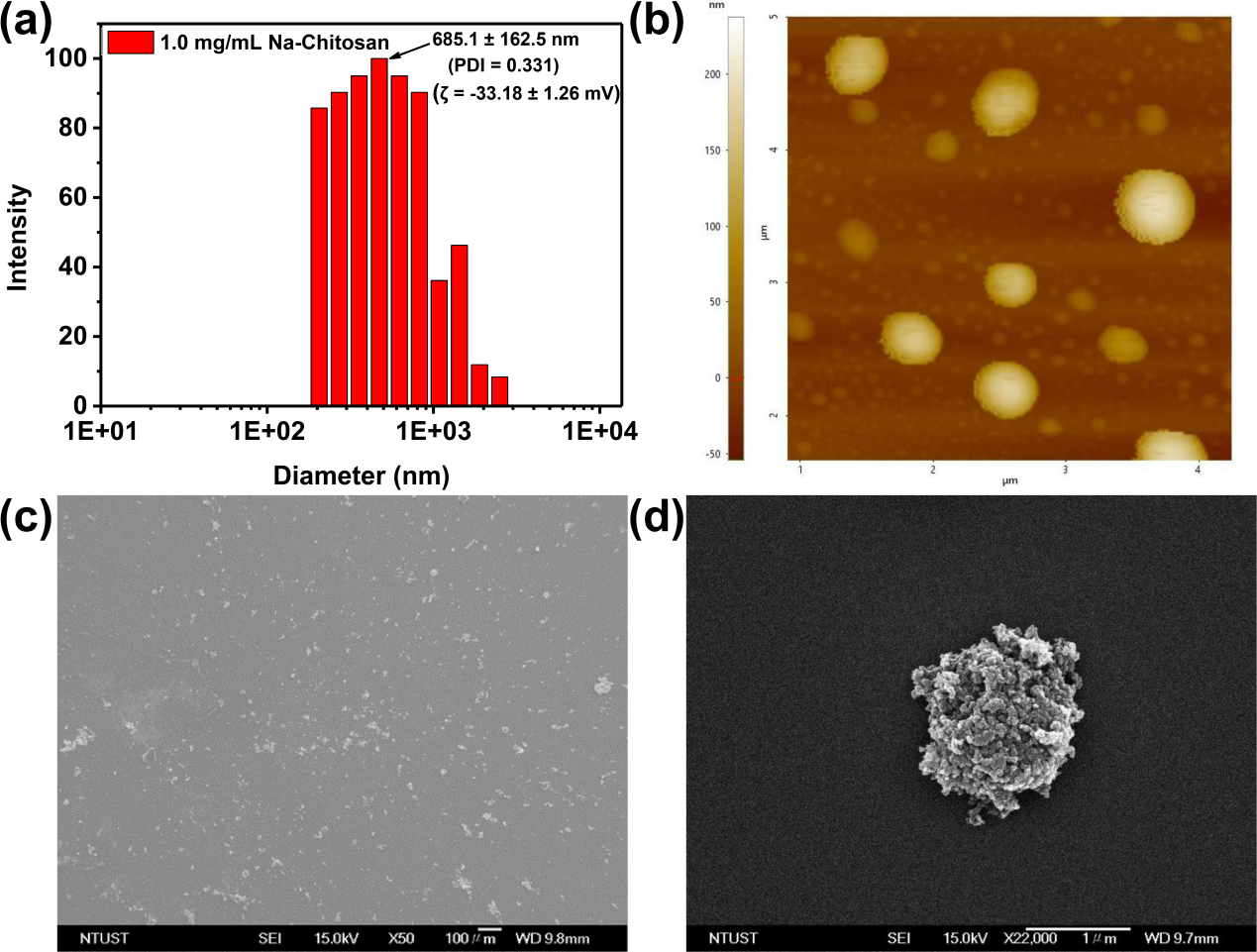


**Figure S7: (a)** DLS profile for Na-Chitosan (1 mg/mL) in water and **(b)** AFM image and SEM images at **(c)** low and **(d)** high magnification of spin-coated Na-Chitosan recorded at 25 °C.

**
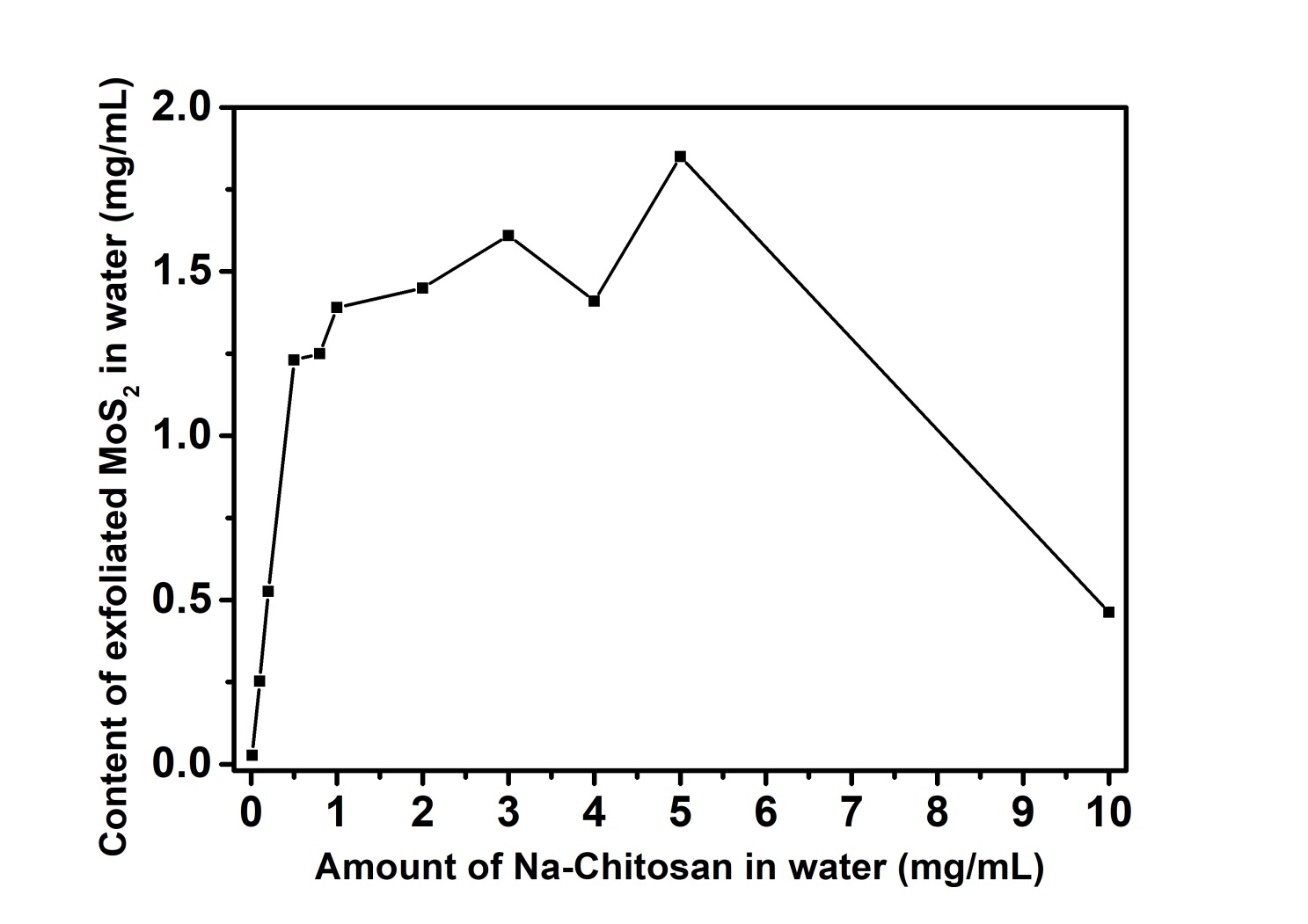
**

**Figure S8:** Relationship between the amount of Na-Chitosan and the content of MoS_2_ exfoliated in water.

**
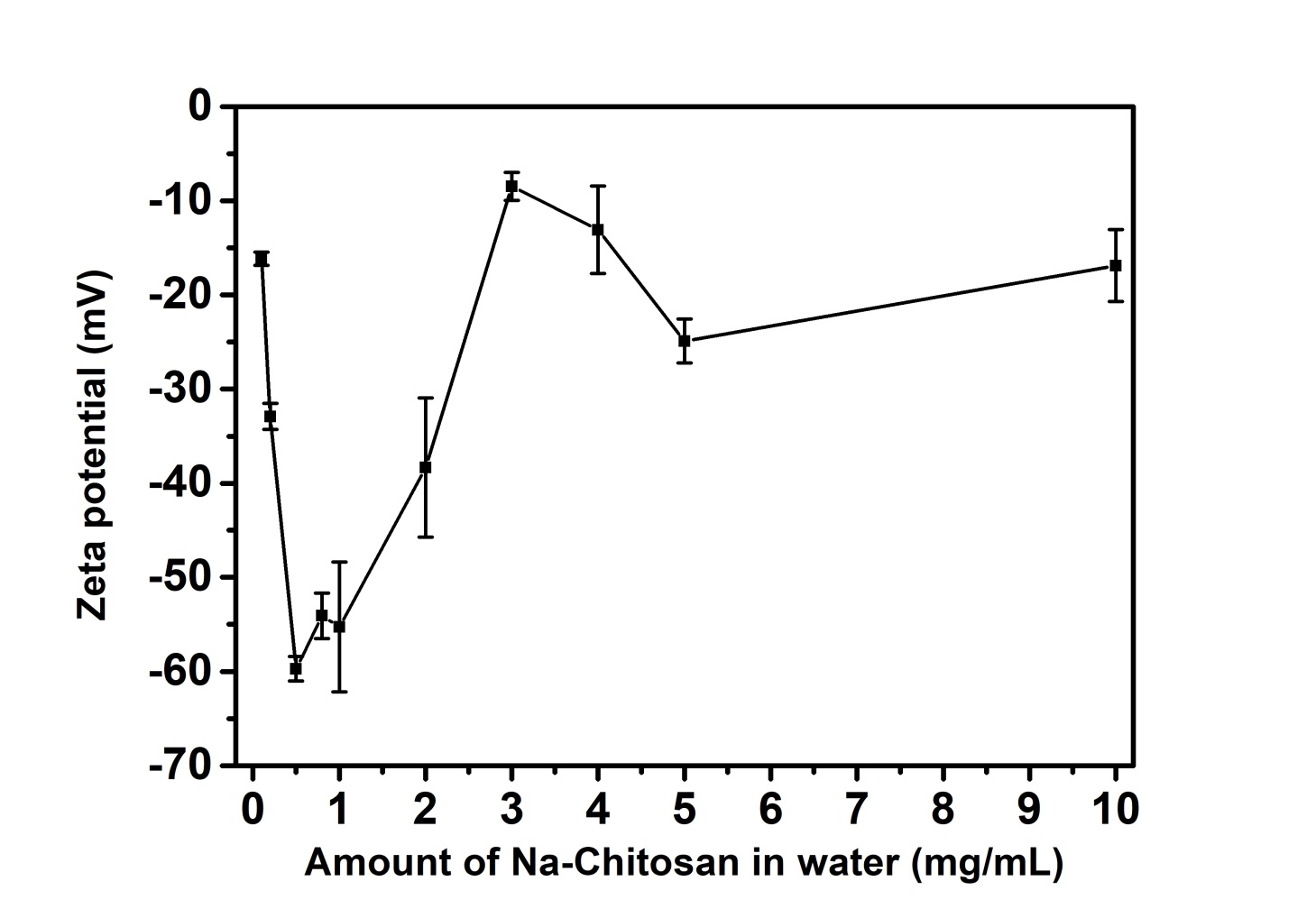
**

**Figure S9:** Relationship between the amount of Na-Chitosan and the zeta potential values of MoS_2_ exfoliated in water.


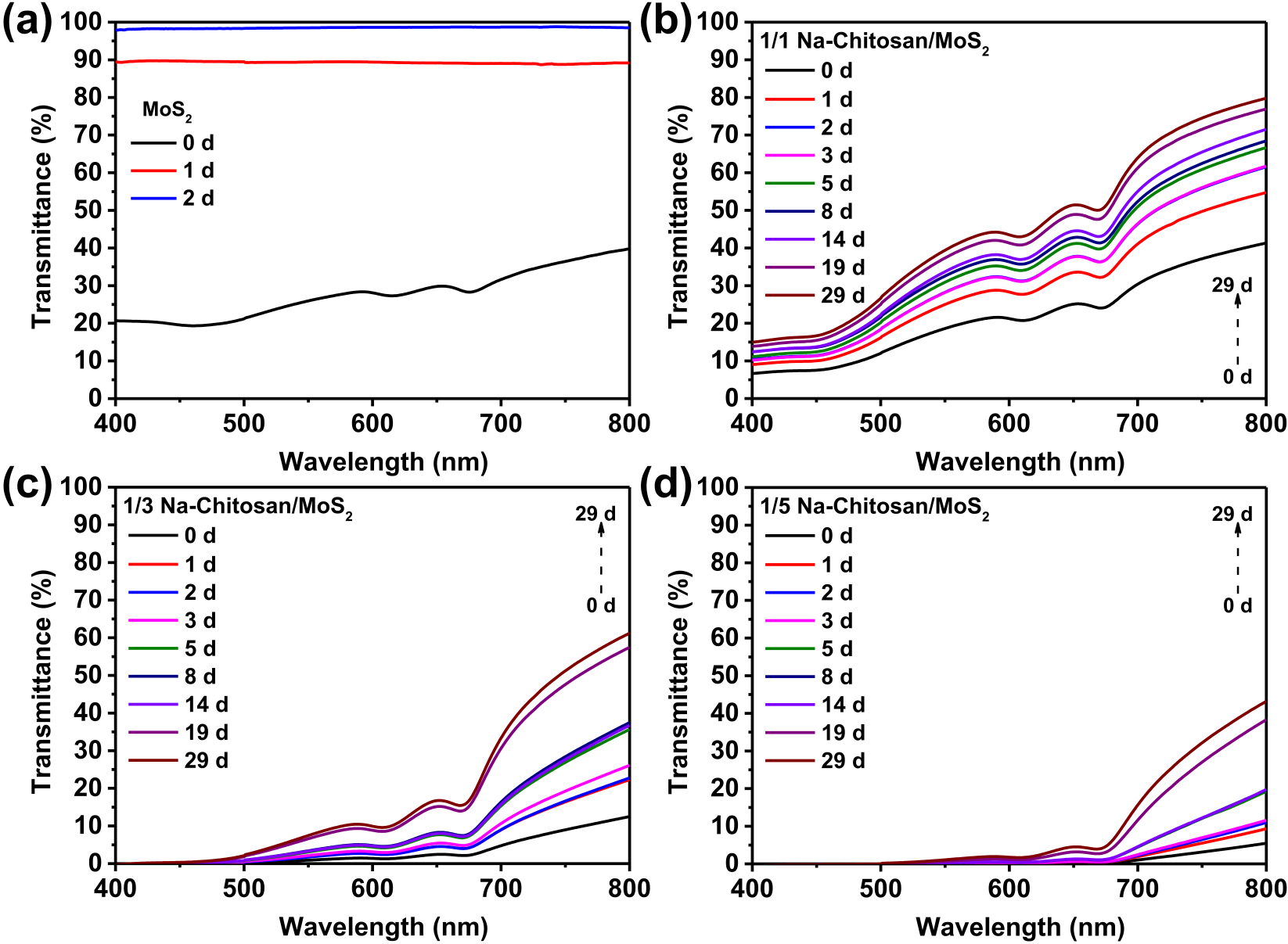


**Figure S10:** Time-dependent transmittance plots of **(a)** pristine MoS_2_ and **(b)** 1/1, **(c)** 1/3, and **(d)** 1/5 Na-Chitosan/MoS_2_ in aqueous solutions with pH 7.4.


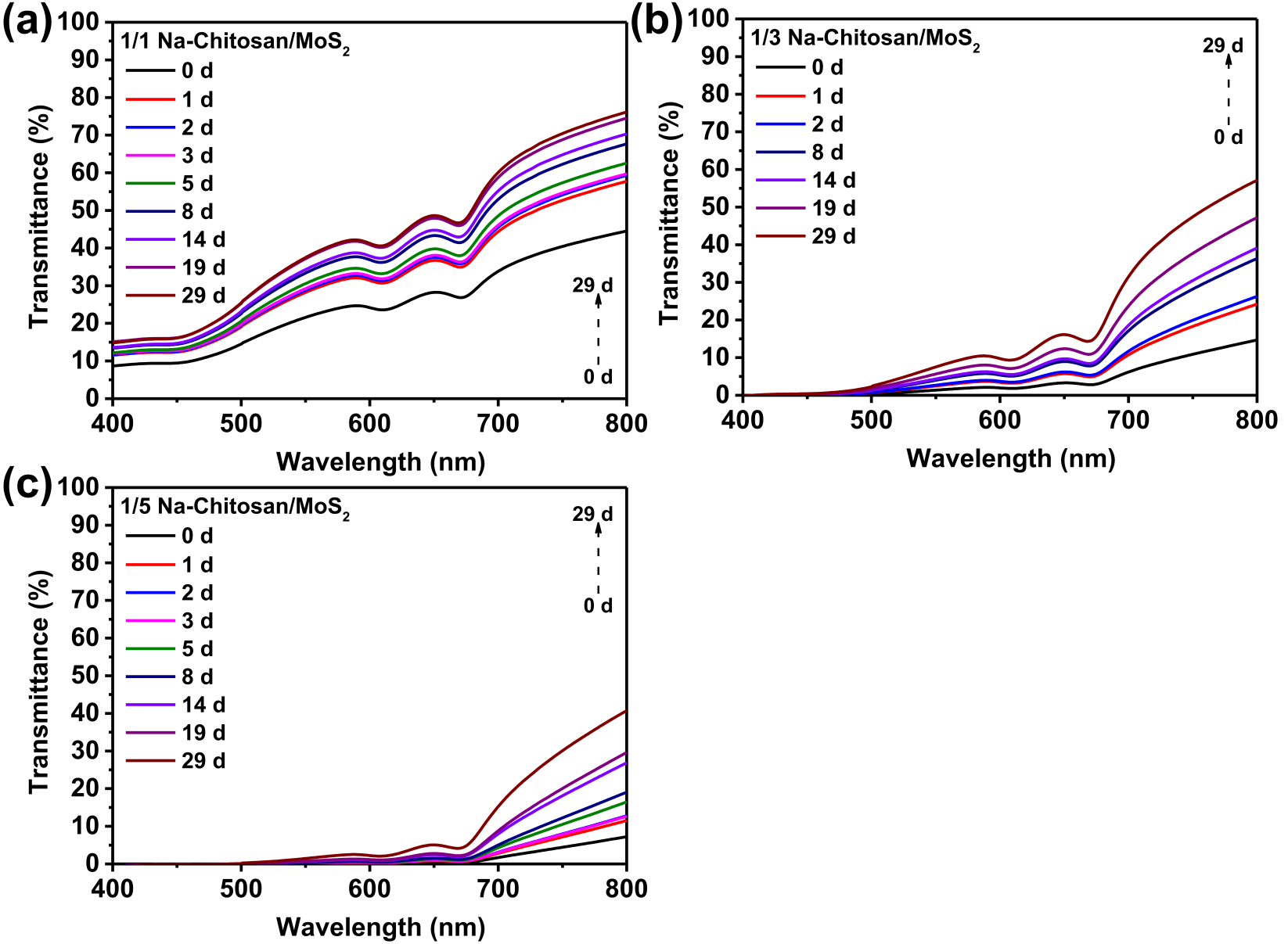


**Figure S11:** Time-dependent transmittance plots of **(a)** 1/1, **(b)** 1/3, and **(c)** 1/5 Na-Chitosan/MoS_2_ in aqueous solutions with pH 1.0.

**
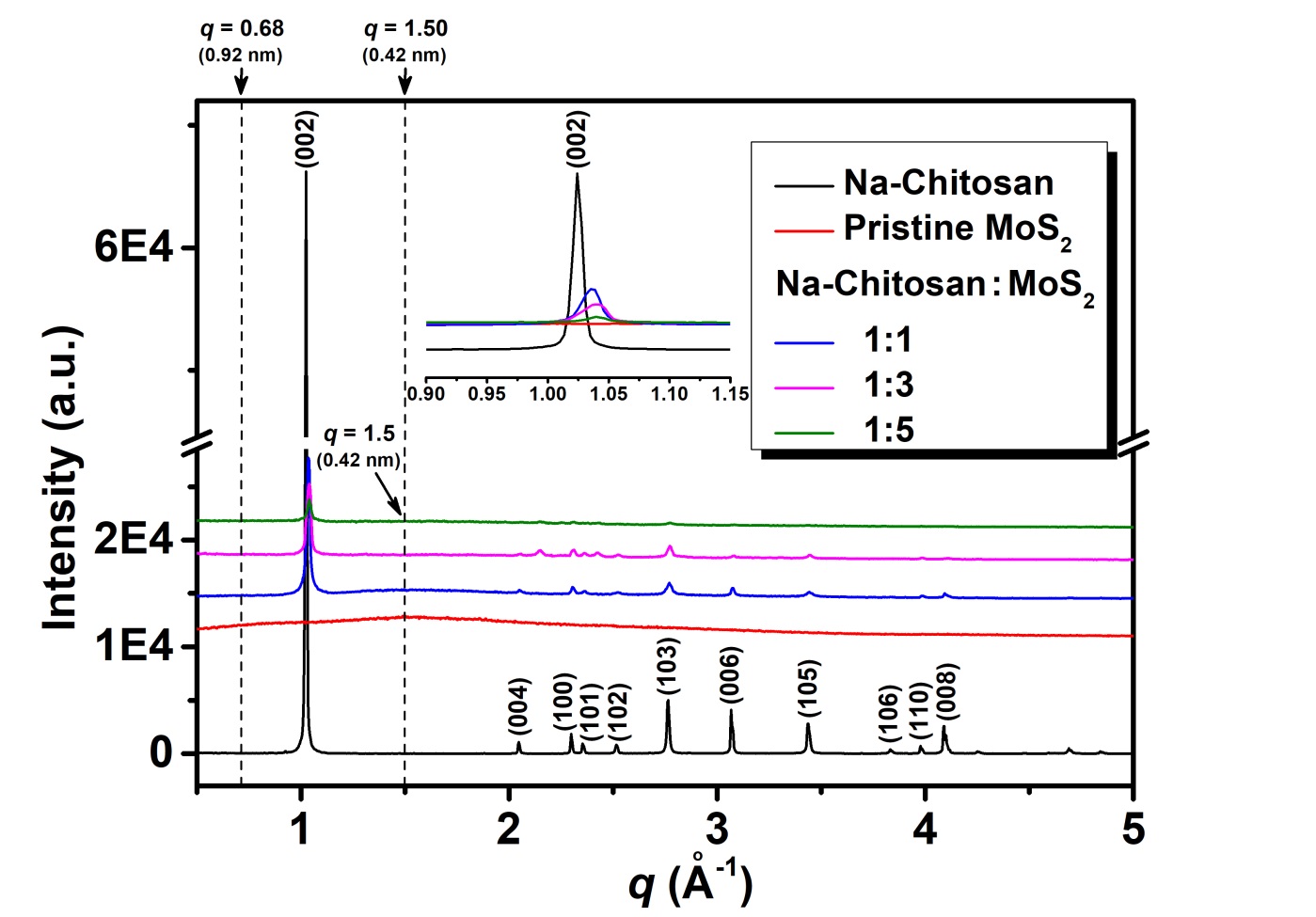
**

**Figure S12:** WAXS profiles of pristine MoS_2_, Na-Chitosan, and the 1/1, 1/3, and 1/5 Na-Chitosan/MoS_2_ composites obtained at 25 °C. The inset in the upper middle depicts an enlarged view of the (002) peak observed in the region of *q* = 0.9-1.15 Å^-1^.

**
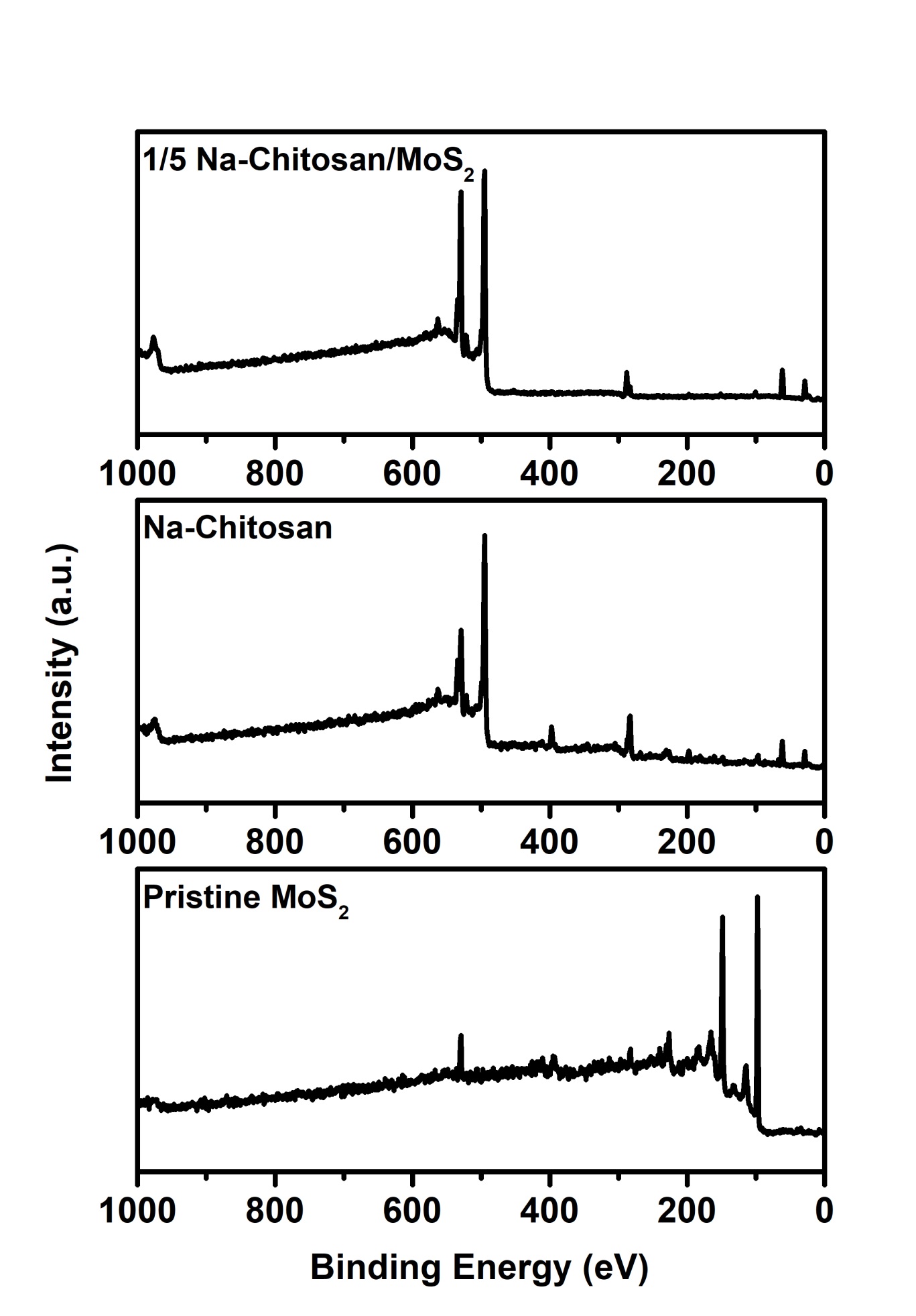
**

**Figure S13:** Full XPS spectra for pristine MoS_2_, Na-Chitosan, and 1/5 Na-Chitosan/MoS_2_ composites.


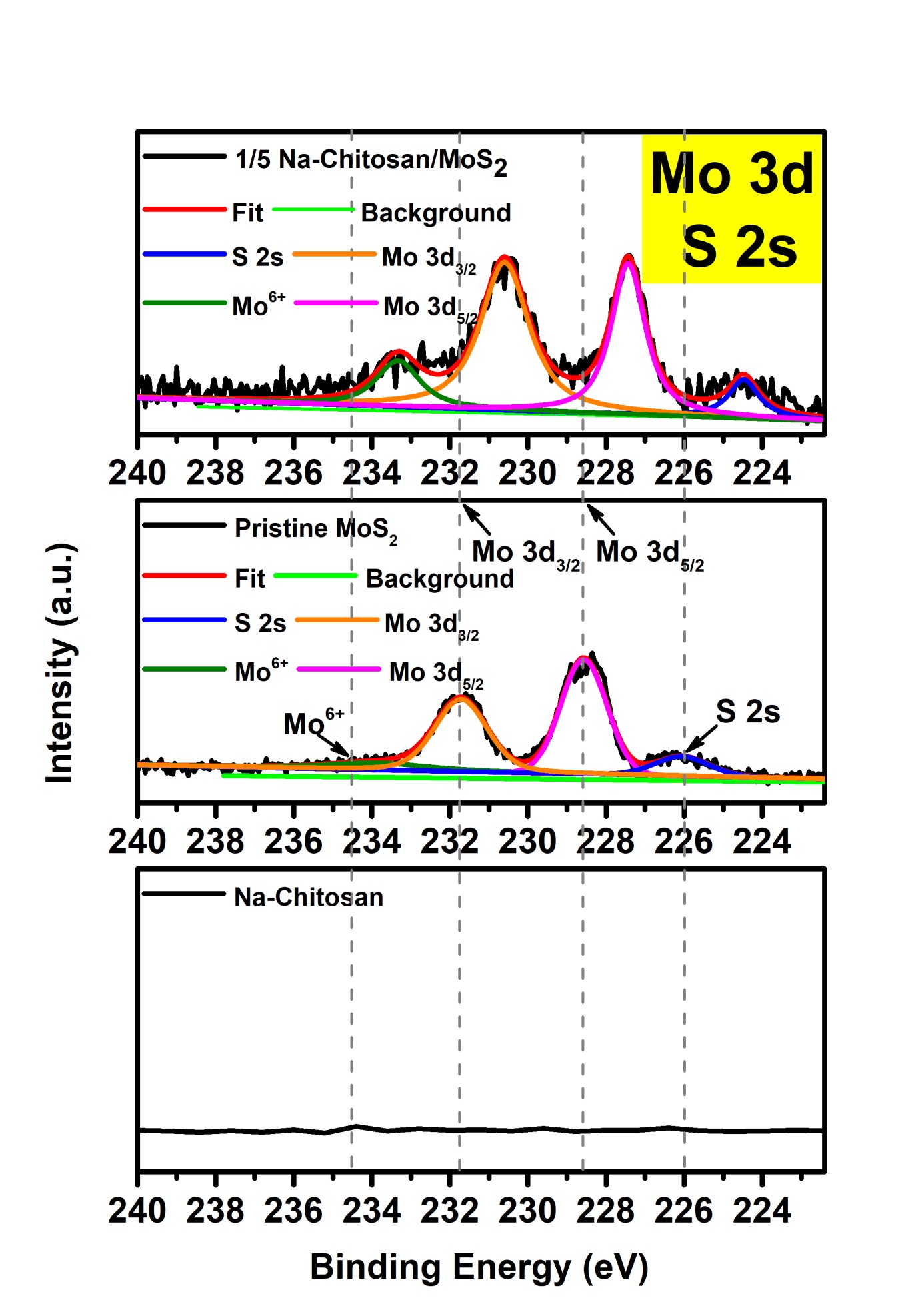


**Figure S14:** XPS spectra of Mo 3d/S 2s of pristine MoS_2_, Na-Chitosan, and 1/5 Na-Chitosan/MoS_2_ composites.


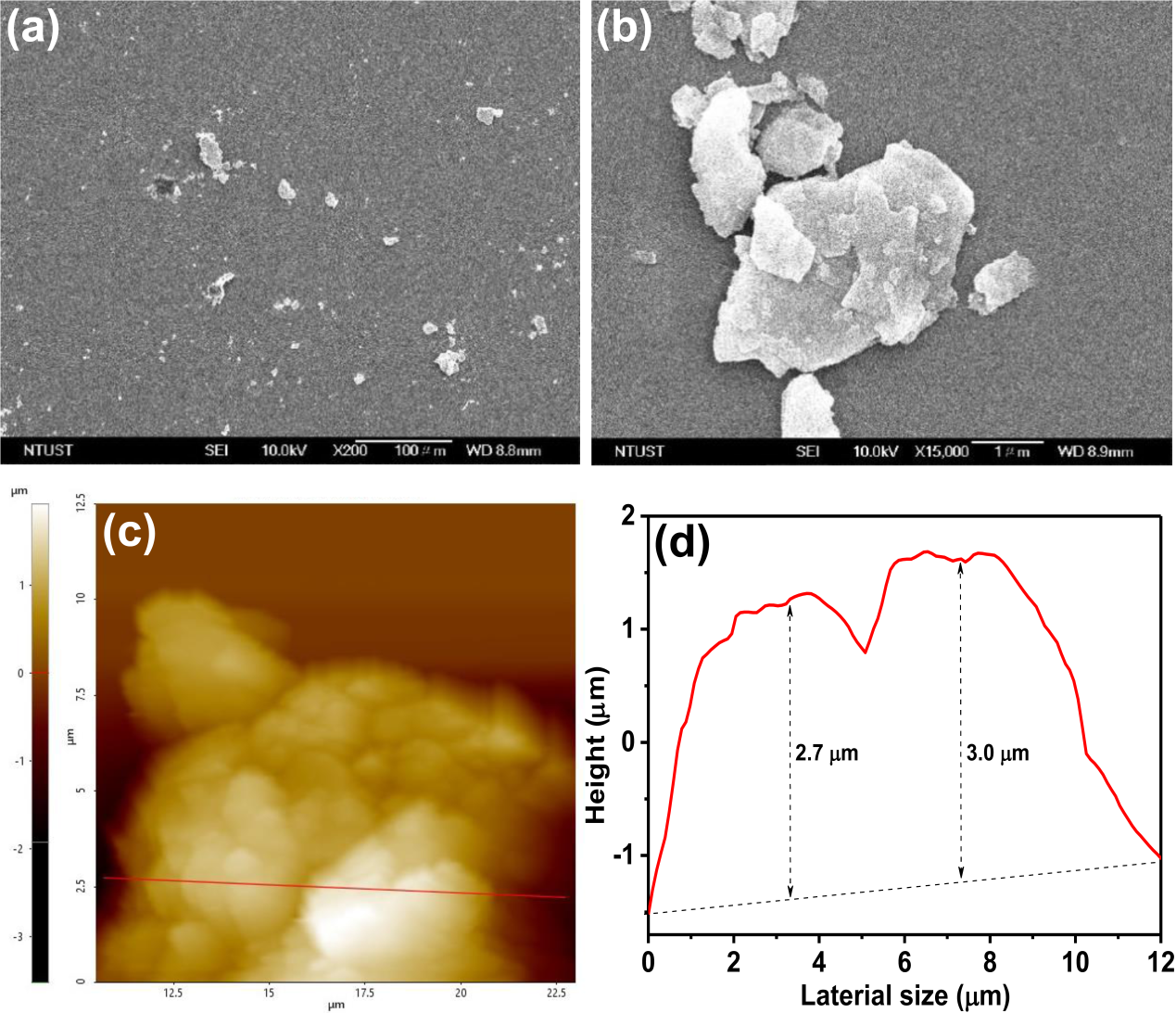


**Figure S15:** SEM images at **(a)** low and **(b)** high magnification of pristine MoS_2_. **(c)** AFM image and **(d)** topographical profile of pristine MoS_2_ measured at 25 °C. **(d)** Line contours corresponding to the red line drawn within the AFM image in (c), representing the lateral and height distributions of the pristine MoS_2_.


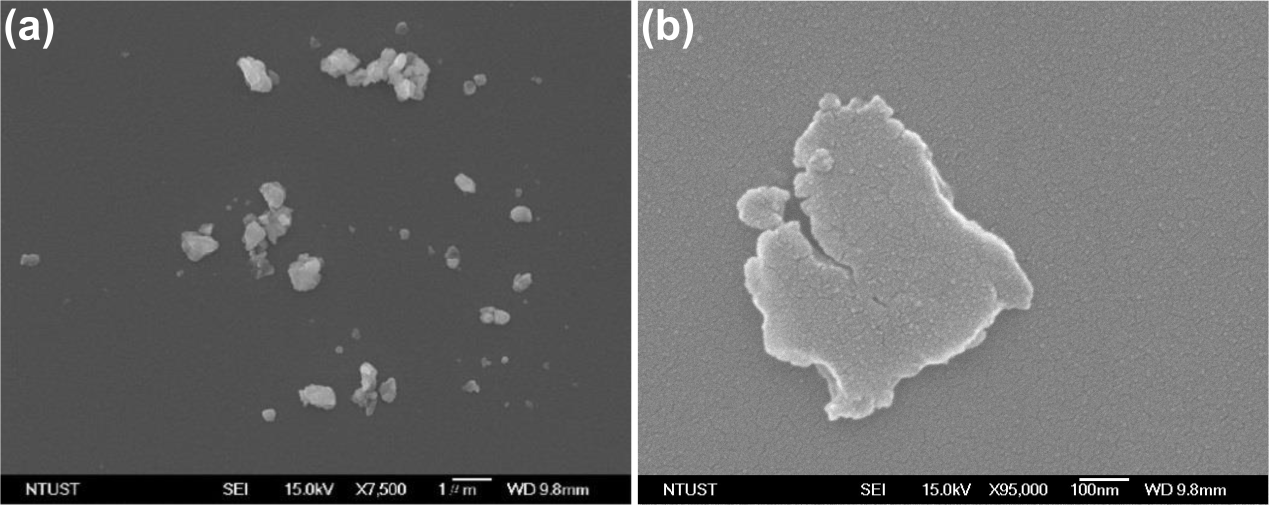


**Figure S16:** SEM images at **(a)** low and **(b)** high magnification of spin-coated 1/5 Na-Chitosan/MoS_2_ composites.


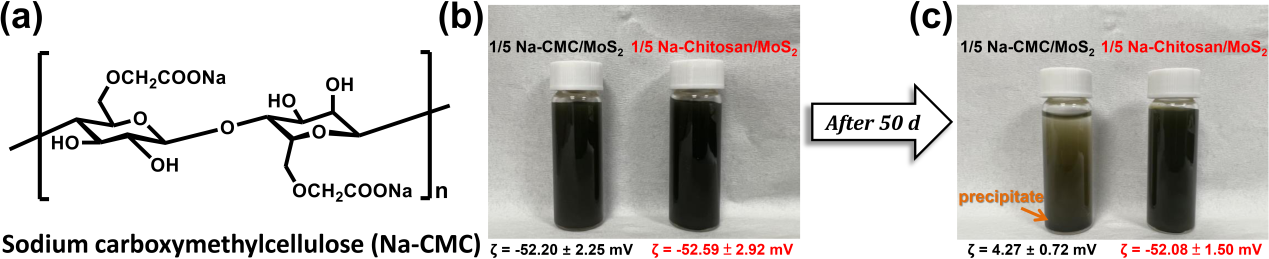


**Figure S17:** **(a)** Chemical structure of Na-CMC. Macroscopic photographs and zeta potential values of 1/5 Na-CMC/MoS_2_ and 1/5 Na-Chitosan/MoS_2_ aqueous solutions **(b)** after completion of preparation and **(c)** after 50 d of storage at room temperature (at approximately 20‒25 °C).


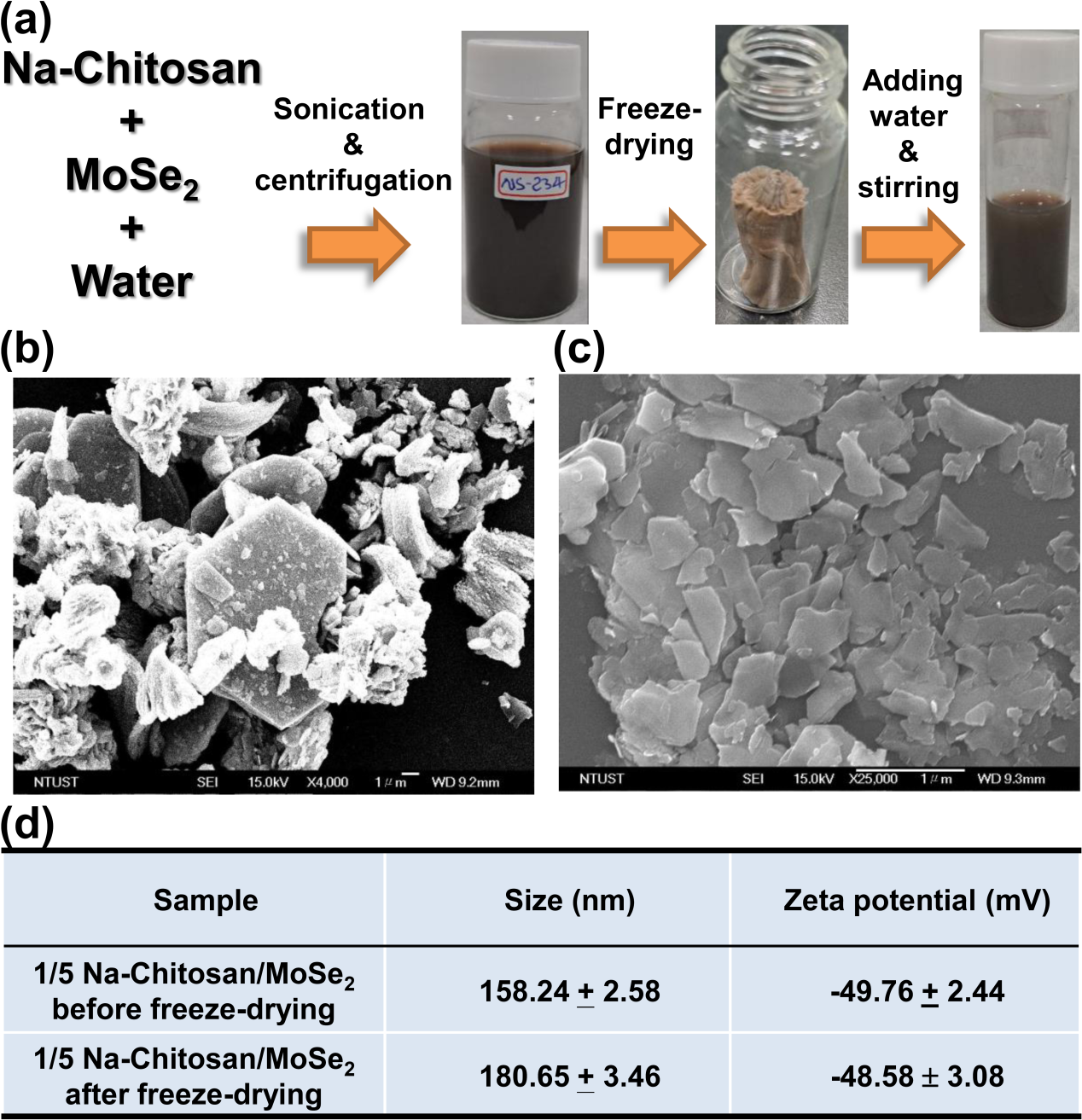


**Figure S18: (a)** Process of exfoliating MoSe_2_ using Na-Chitosan and re-dispersion of the freeze-dried solid in water. SEM images of **(b)** pristine MoSe_2_ and **(c)** 1/5 Na-Chitosan/MoSe_2_ composites. **(d)** Particle size and zeta potential values of 1/5 Na-Chitosan/MoSe_2_ aqueous solution before and after freeze-drying.


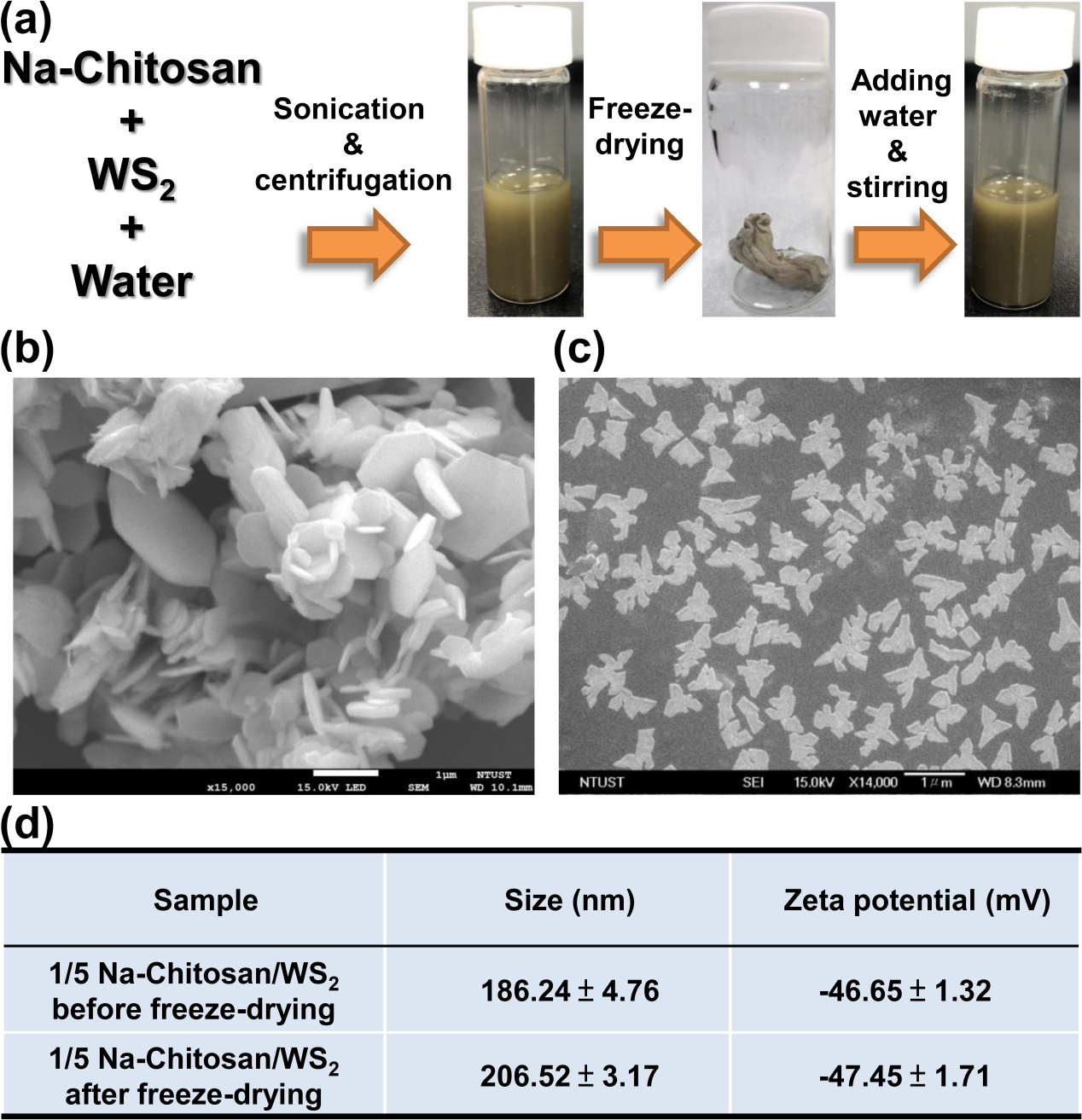


**Figure S19: (a)** Process of exfoliating WS_2_ using Na-Chitosan and re-dispersion of the freeze-dried solid in water. SEM images of **(b)** pristine WS_2_ and **(c)** 1/5 Na-Chitosan/WS_2_ composites. **(d)** Particle size and zeta potential values of 1/5 Na-Chitosan/WS_2_ aqueous solution before and after freeze-drying.


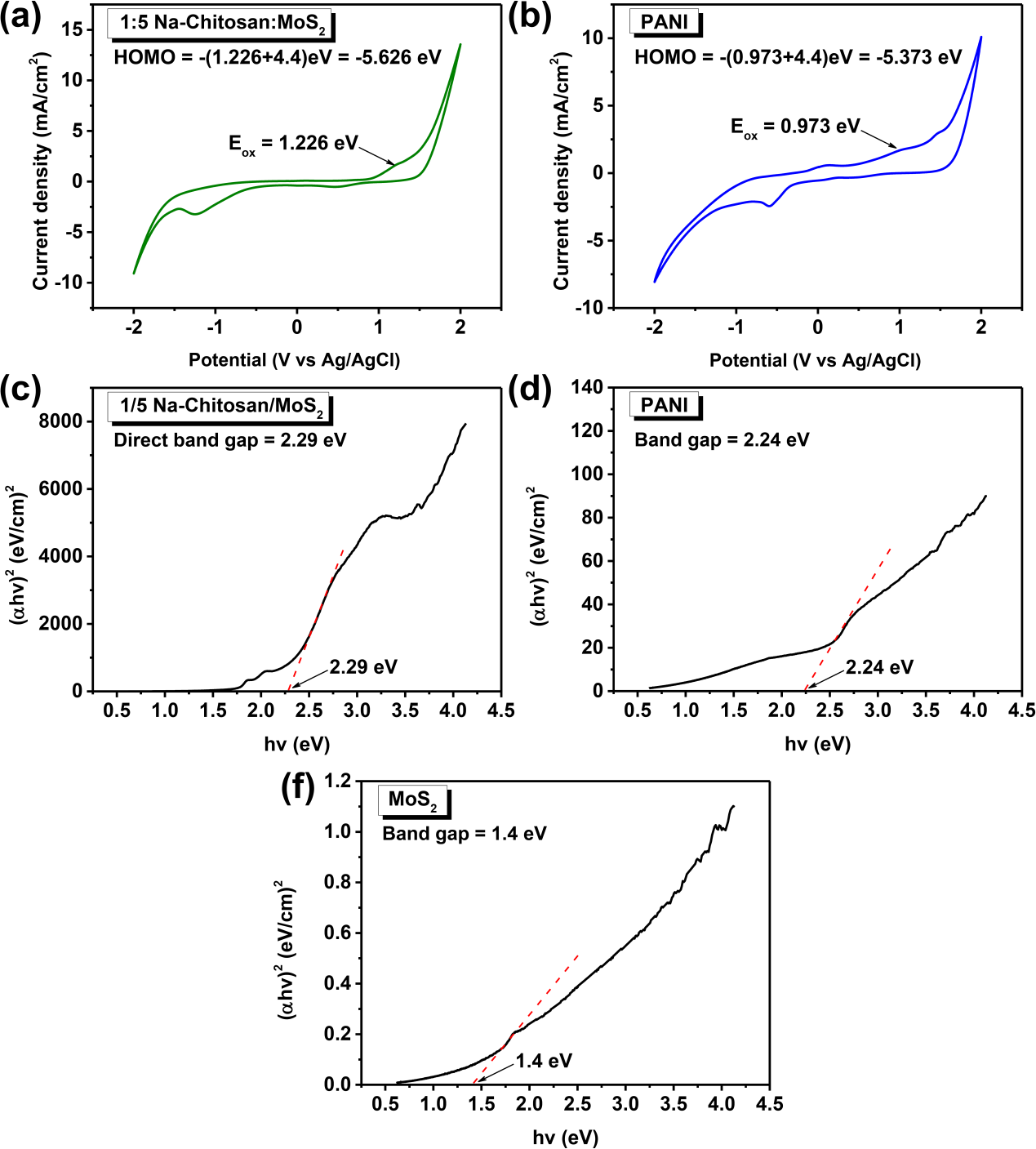


**Figure S20:** CV curves of **(a)** 1/5 Na-Chitosan/MoS_2_ and **(b)** PANI at a scan rate of 50 mV/sec. Tauc's plots for the calculation of *E*_g_ for **(c)** 1/5 Na-Chitosan/MoS_2_, **(d)** PANI, and **(e)** pristine MoS_2_.


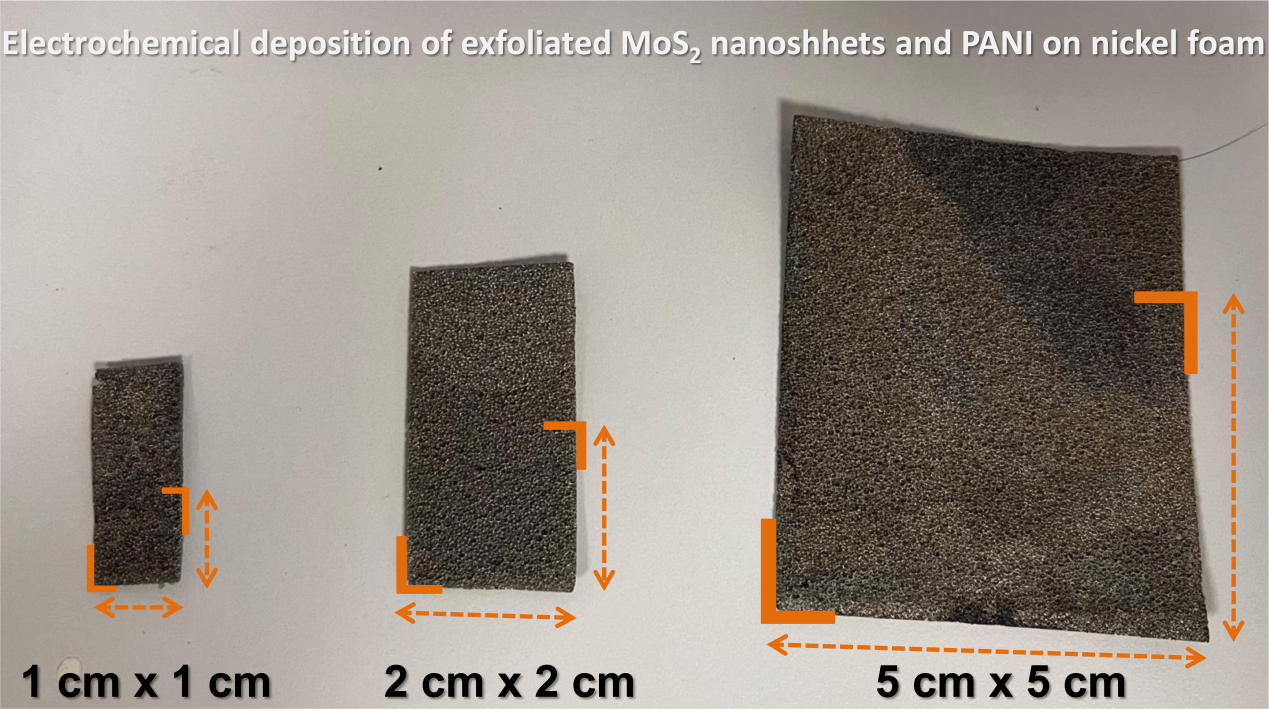


**Figure S21:** Photograph of different sizes of Na-Chitosan/MoS_2_/PANI/NF electrodes prepared through the EP process.


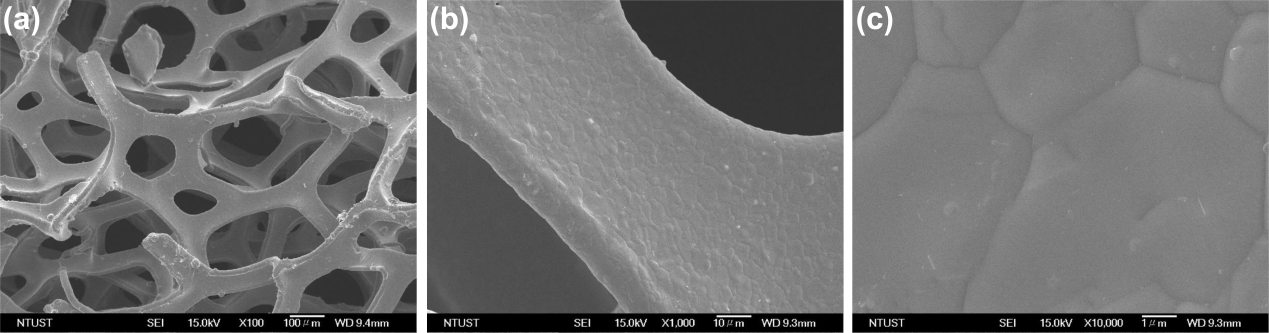


**Figure S22:** SEM images of original NF at **(a)** 100x, **(b)** 1,000x, and **(c)** 10,000x magnification.

**
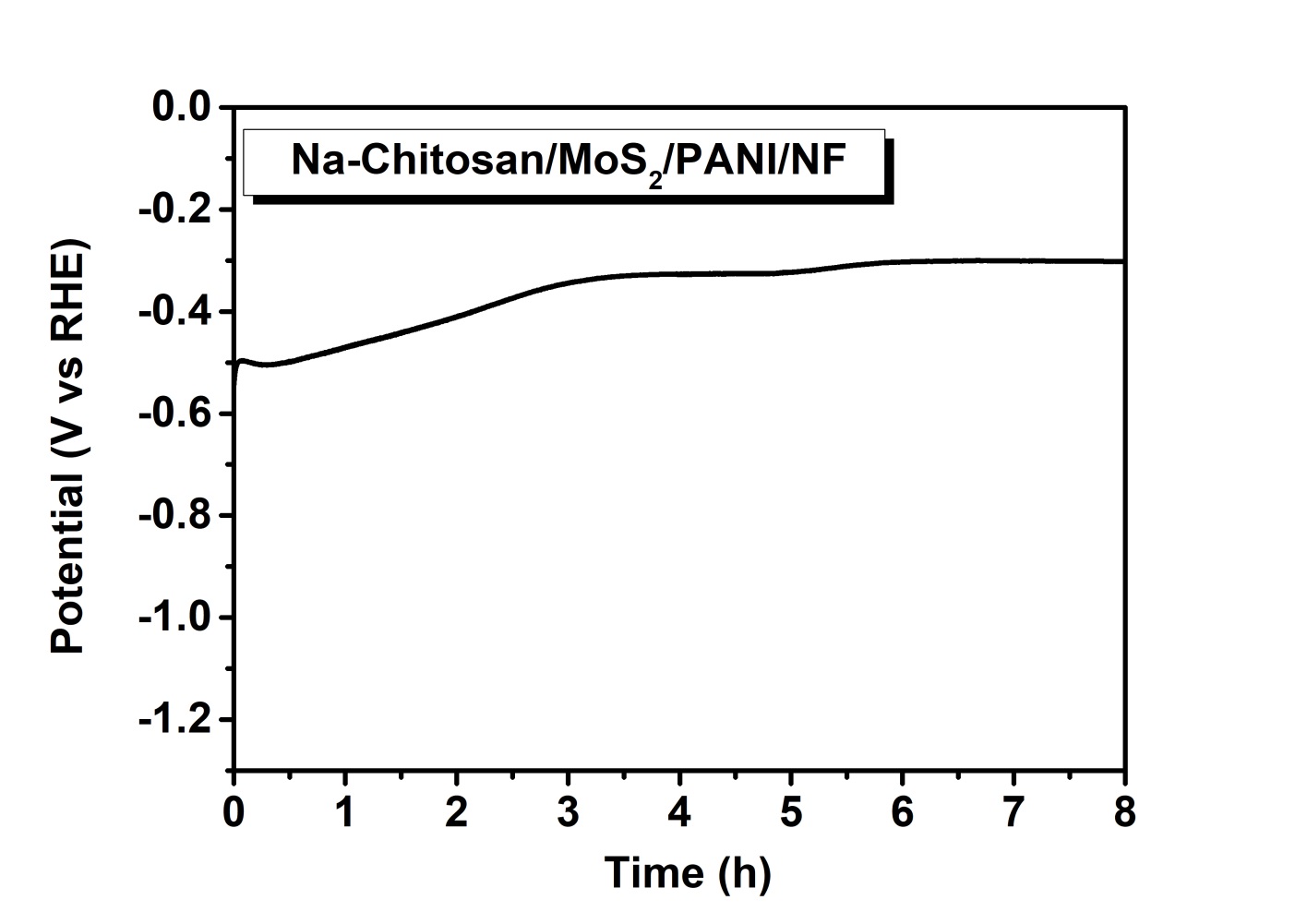
**

**Figure S23:** Chronopotentiometric stability of the Na-Chitosan/MoS_2_/PANI/NF electrodes (after EP process) in 0.5 M H_2_SO_4_ solution for 8 h at a fixed current of 500 mA.

**
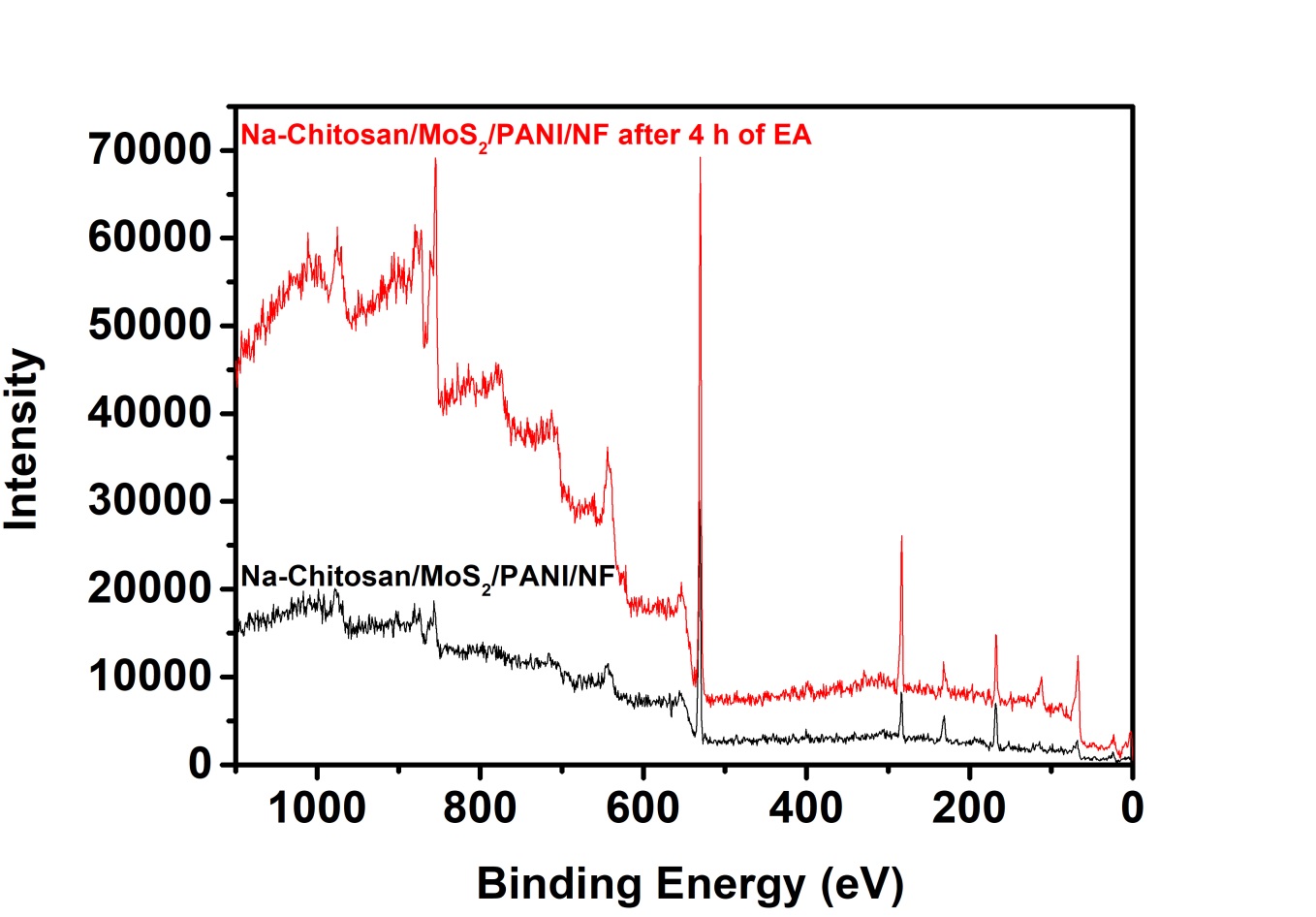
**

**Figure S24:** Full XPS spectra for the Na-Chitosan/MoS_2_/PANI/NF electrodes before and after EA treatment.


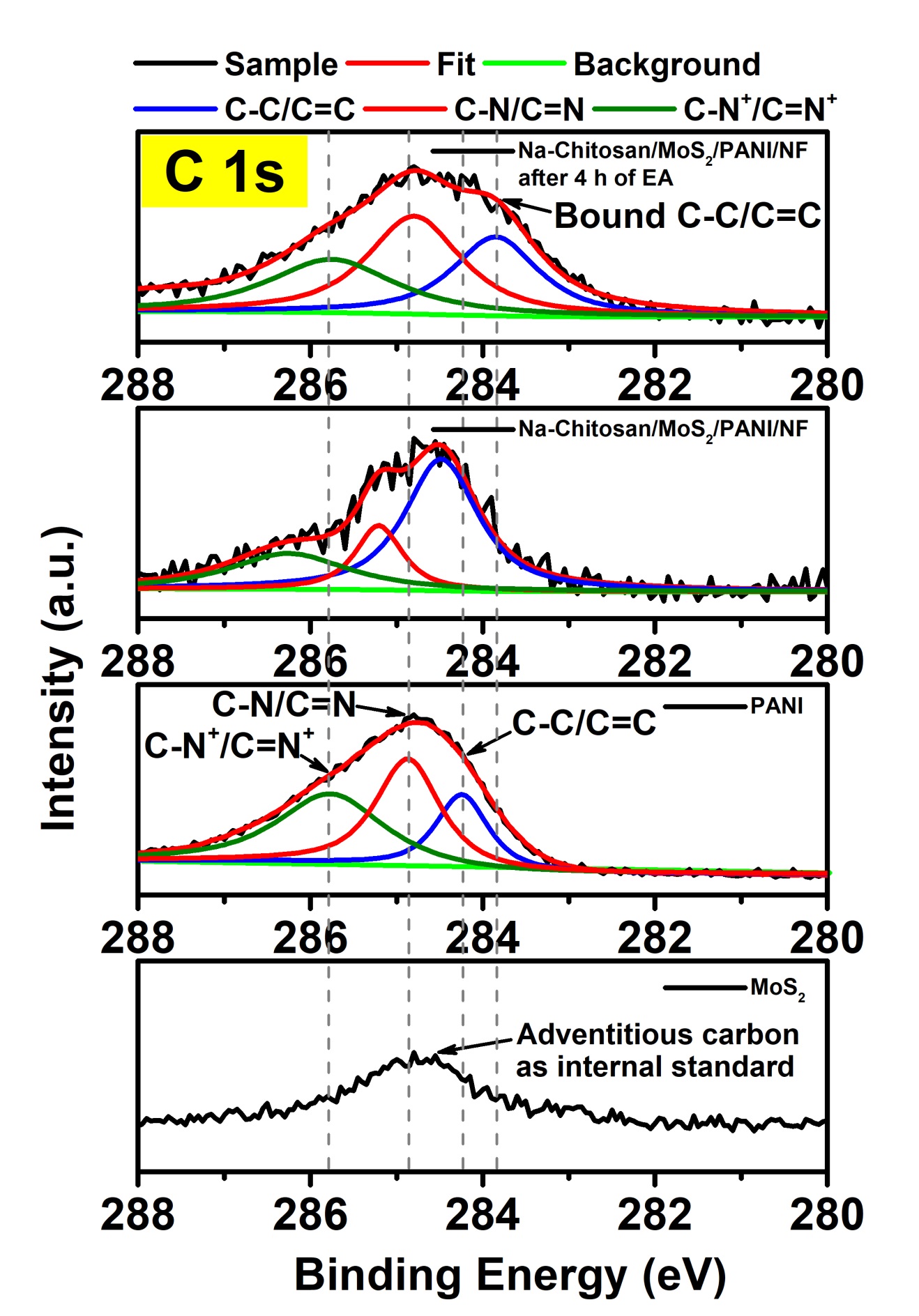


**Figure S25:** XPS spectra of C 1s of pristine MoS_2_, PANI, and 1/5 Na-Chitosan/MoS_2_/PANI/NF electrodes before and after EA treatment.


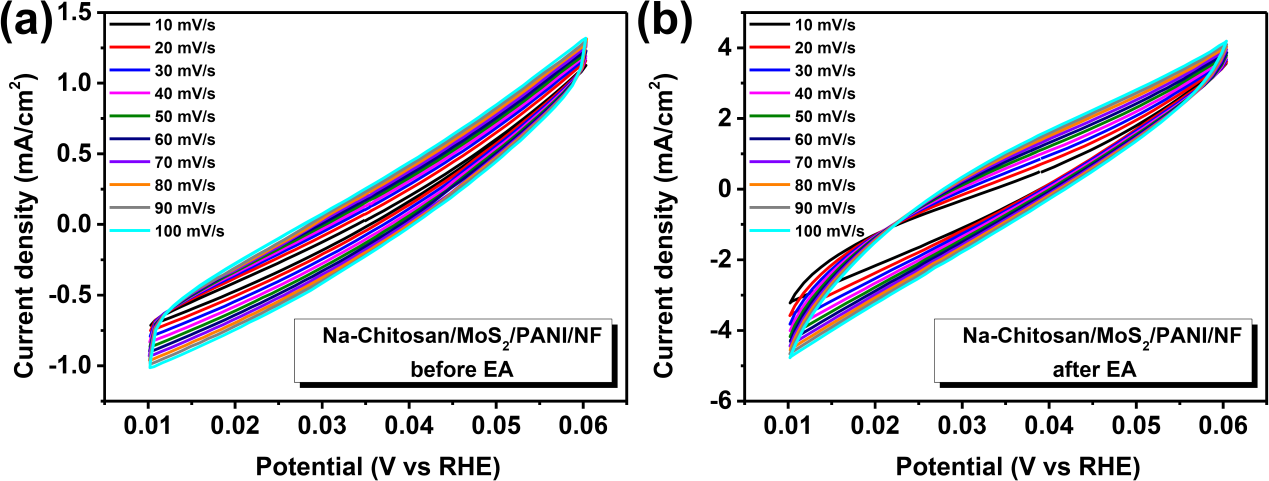


**Figure S26:** *C*_dl_ measurements: CV results for the Na-Chitosan/MoS_2_/PANI/NF electrodes **(a)** before and **(b)** after EA treatment over the scanning range of 0.01−0.06 V at various scan rates in aqueous 0.5 M H_2_SO_4_.


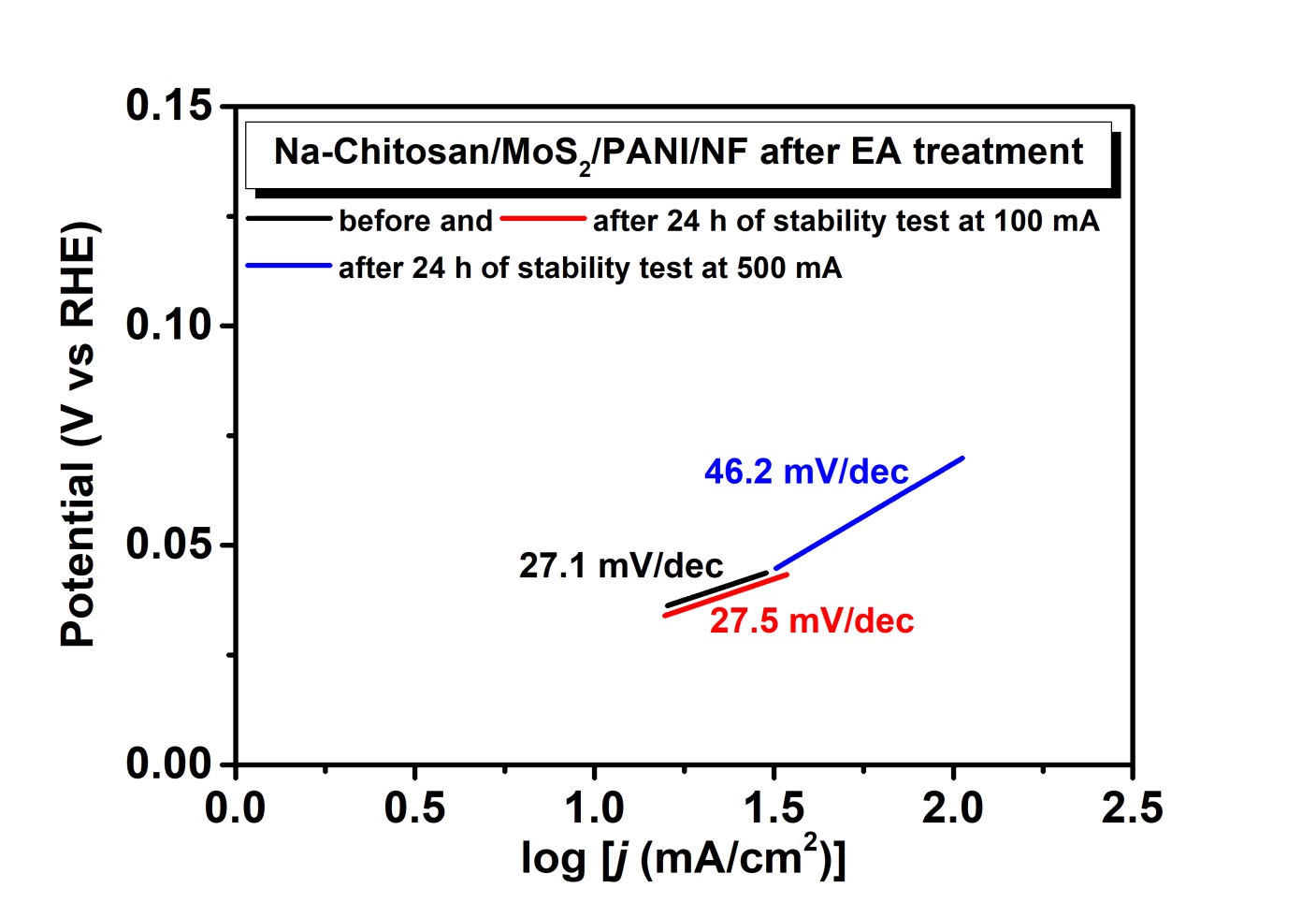


**Figure S27:** Tafel plots of Na-Chitosan/MoS_2_/PANI/NF electrodes before and after 24 h stability testing at 100 mA or 500 mA.


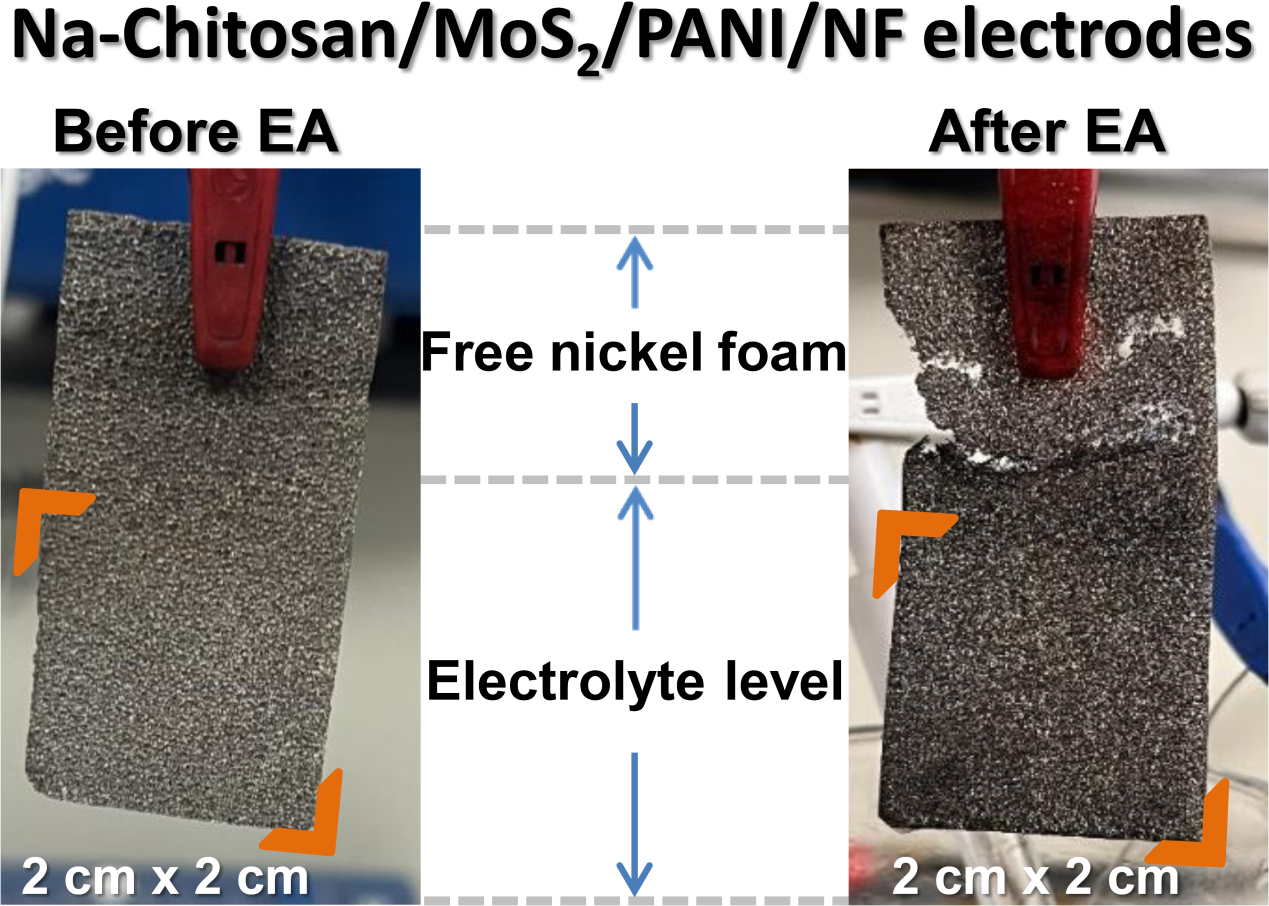


**Figure S28:** Photograph of 2 cm × 2 cm Na-Chitosan/MoS_2_/PANI/NF electrodes before and after EA treatment.

**Table S1:** Calculation of the degree of substitution on the structure of Na-Chitosan.

| **Position of Chitosan** | **DS** | **Chitosan DD (%)** | **Chitosan DA (%)** |
| --- | --- | --- | --- |
| **O-C6(*f6*)** | 0.615 | 97.8 | 2.2 |
| **O-C3(*f3*)** | 0.337 |  |  |
| **N-C2(*f2*)** | 0.168 |  |  |
| **Total DS** | 1.120 |  |  |

**Table S2:** Comparison of the exfoliation time, exfoliated MoS_2_ content (yield), and particle size of Na-Chitosan/MoS_2_ system with values reported in the literature.

| **Exfoliation method** | **Surfactant (Yes/No)** | **Exfoliation time (h)** | **Yield (g/L)** | **Size (nm)** | **Reference** |
| --- | --- | --- | --- | --- | --- |
| Liquid exfoliation | Yes | 0.5 | 0.25 | 280 | 40 |
| Shear exfoliation | Yes | 10 | 0.4 | 85 | 41 |
| Mechanical & liquid exfoliation | Yes | 16 | 0.8 | 50-700 | 42 |
| Mechanical & liquid exfoliation | No | 1 | 0.14 | 242 | 43 |
| Liquid phase exfoliation & solvothermal functionalization | Yes | 12 | 0.17 | 90 | 44 |
| Liquid exfoliation | Yes | 0.5 | 1.85 | 135 | This work |

**Table S3:** Comparison of the overpotential, Tafel slope, and impedance results for Na-Chitosan/MoS_2_/PANI/NF electrodes before and after EA treatment, PANI/NF, and 20 wt% Pt/C.

|  | **Na-Chitosan/MoS_2_/PANI/NF**  **before EA treatment** | **Na-Chitosan/MoS_2_/PANI/NF after EA treatment** | **PANI/NF** | **NF** | **20 wt% Pt/C** |
| --- | --- | --- | --- | --- | --- |
| **Overpotential** **(mV)**  **at -10 mA/cm^2^** | 37.8 | 14.8 | 253.2 | 461.1 | 2.4 |
| **Tafel slope (mV/dec)** | 112.1 | 26.3 | 158.3 | 296.9 | 22.8 |
| **Resistance (ohm)** | 7.9 | 1.7 | 30.1 | 102.5 | 3.3 |

**Movie S1:** Redispersibility of exfoliated Na-Chitosan/MoS_2_ powder after freeze drying in aqueous solution at 25 °C (see **Supplementary Video S1** for more details).
